# A Critical Review of Resistance and Oxidation Mechanisms of Sb-Oxidizing Bacteria for the Bioremediation of Sb(III) Pollution

**DOI:** 10.3389/fmicb.2021.738596

**Published:** 2021-09-07

**Authors:** Renjian Deng, Yilin Chen, Xinpin Deng, Zhongjie Huang, Saijun Zhou, Bozhi Ren, Guizhong Jin, Andrew Hursthouse

**Affiliations:** ^1^School of Civil Engineering, Hunan University of Science and Technology, Xiangtan, China; ^2^Hunan 402 Geological Prospecting Part, Changsha, China; ^3^Hsikwangshan Twinkling Star Co., Ltd., Lengshuijiang, China; ^4^School of Computing, Engineering and Physical Sciences, The University of the West of Scotland, Paisley, United Kingdom

**Keywords:** Sb-oxidizing bacteria, antimony, Sb(III)-resistance, oxidation, mechanism

## Abstract

Antimony (Sb) is a priority pollutant in many countries and regions due to its chronic toxicity and potential carcinogenicity. Elevated concentrations of Sb in the environmental originating from mining and other anthropogenic sources are of particular global concern, so the prevention and control of the source of pollution and environment remediation are urgent. It is widely accepted that indigenous microbes play an important role in Sb speciation, mobility, bioavailability, and fate in the natural environment. Especially, antimony-oxidizing bacteria can promote the release of antimony from ore deposits to the wider environment. However, it can also oxidize the more toxic antimonite [Sb(III)] to the less-toxic antimonate [Sb(V)], which is considered as a potentially environmentally friendly and efficient remediation technology for Sb pollution. Therefore, understanding its biological oxidation mechanism has great practical significance to protect environment and human health. This paper reviews studies of the isolation, identification, diversity, Sb(III) resistance mechanisms, Sb(III) oxidation characteristics and mechanism and potential application of Sb-oxidizing bacteria. The aim is to provide a theoretical basis and reference for the diversity and metabolic mechanism of Sb-oxidizing bacteria, the prevention and control of Sb pollution sources, and the application of environment treatment for Sb pollution.

## Introduction

Antimony (Sb) is widely present in the lithosphere, hydrosphere, and biosphere in very trace amount. Due to its chronic toxicity, potential carcinogenicity, and global migration (Henríquez-Hernández et al., 2017; Herath et al., 2017), Sb and its compounds are classified as priority pollutants by many countries and organizations, such as the European Council, the United States Environmental Protection Organization, the Chinese Environmental Protection Agency, and the Japanese Environmental Protection Agency (Council of the European Union, 1990; United States Environmental Protection Agency, 1997; China National Standardization Administration Commission, 2006). The permitted maximum concentration of Sb in drinking water has been set by the World Health Organization at 5 μg/L (Shan et al., 2014). Against this standard, many rivers and groundwater zones in China are seriously polluted, especially in the vicinity of Sb mining area.

The element Sb is a national priority as one of the significant resources widely used in semiconductors, diode, glass, flame retardant, and other industries with high international demand (He et al., 2019). Anthropogenic activities such as mining and smelting industries are the main source of Sb contamination (Henríquez-Hernández et al., 2017; Herath et al., 2017). Approximately 140,000 tons of Sb is mined and supplied to various industrial sectors annually (Filella et al., 2002; Shan et al., 2014; Li J. et al., 2018). The large volumes of waste rocks from mining are continuously weathered at the surface and release antimony ions under the interaction of chemistry and microorganism (Palmer et al., 2019; Ren et al., 2019), which have elevated the content of Sb in water and soil environment (Kuang et al., 2013; Wang et al., 2019), posing a great risk to the aquatic ecosystems and to drinking water supply, agricultural security, and human health particularly close to the mine site (He et al., 2002, 2012). Consequentially since the 1970s, much attention has been paid to validating techniques for Sb removal. Numerous high-efficiency removal technologies and methods, such as coagulation precipitation, electrochemistry, ion exchange, adsorption, and membrane filtration (Zhang et al., 2019; Hassan et al., 2020; Long et al., 2020; Wang et al., 2020; Xiong et al., 2020; Zeng et al., 2020), have been developed to treat aqueous solutions and support the phytoremediation of Sb-contaminated soil. A crucial aspect of the geochemical behavior of Sb in nature is the dissolution and oxidation of antimonite from Sb-contaminated minerals and rocks (Wilson et al., 2010; Wang X. Q. et al., 2011), which are caused by coupling of mining activities with microbial interaction. However, the role of microbes and the detailed molecular mechanism in the dissolution and oxidation of antimonite in Sb-containing minerals and pristine rocks remain poorly understood.

**FIGURE 1:**
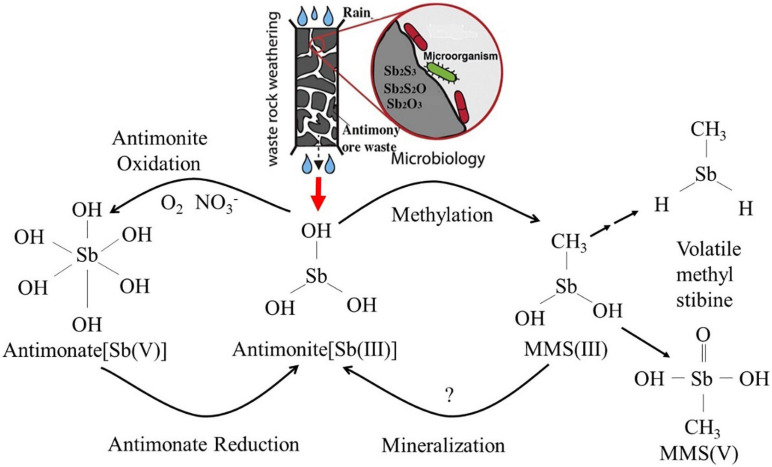
The biotransformation relationship among the various forms in Sb biogeochemical cycling (Li, 2017).

As a global priority pollutant, the toxicity and mobility of Sb are dependent upon its chemical speciation in the environment. Elemental Sb is more toxic than its salts, and inorganic species of Sb are more toxic than organic species (Zhang et al., 2010; Shtangeeva et al., 2011). Moreover, the toxicity of antimonite [Sb(III)] is orders of magnitude more than that of antimonate [Sb(V)] (He et al., 2019). The chemical properties of Sb have aroused greater interest as its biogeochemical behavior in the environment is further studied. The abiotic dissolution and oxidation of Sb(III) from antimonite, antimony blende, and valentinite are very slow at neutral conditions (Ren et al., 2017, 2018; He et al., 2019). However, this process is significantly accelerated under microbial-mediated condition (Levett et al., 2020; Loni et al., 2020), implying that it is the biotic dissolution and oxidation that contribute to the formation of Sb pollution (Bagherifam et al., 2019). Sb is highly toxic to organisms, but some indigenous microbes can survive in highly Sb-contaminated environments, even subsisting on oxidized antimony-containing minerals as energy sources. Under continuous stress from high concentrations of Sb, these microbes have evolved various metabolic reactions, including dissolution, oxidation, reduction, methylation, mineralization, and bioaccumulation (Li, 2017; Figure 1). These mechanisms play a vital role in the geochemical cycle of Sb and also determine the ultimate fate of Sb in the environment. Microbial oxidation reactions can produce less-toxic and more mobile Sb(V) (Li et al., 2015; Loni et al., 2020), and it is considered to be an environmentally friendly and efficient method for the remediation of antimony pollution (Filella et al., 2007). It is thus of great significance to understand the oxidation and transformation of Sb and the interaction between microbes and the various forms of Sb under microbial mediation, as these reactions will enhance our understanding of Sb behavior in its biogeochemical cycle and environmental impacts (He and Chen, 2014; Burton et al., 2019). These interactions are also fundamental in developing Sb pollution prevention and control (Fan et al., 2019; Guo et al., 2019; He et al., 2019; Karimian et al., 2019a, b) and bioremediation technology (Shtangeeva et al., 2011; Long et al., 2019; Ma et al., 2019; Sun et al., 2019; Deng et al., 2020). Although the microbial metabolism of Sb has been preliminarily discussed in several studies (Li et al., 2015; Liu et al., 2015), Sb(III) resistance mechanisms; Sb(III) oxidation characteristics and mechanisms; and the interaction between Sb-oxidizing bacteria and Sb are still poorly understood, and bioremediation technology for Sb pollution is difficult to apply in practice (He and Chen, 2014; Burton et al., 2019). Based on this, we review the progress of research on the role of Sb-oxidizing bacteria, focusing on the following: (1) the screening, identification, diversity, and phylogenetic level of Sb-oxidizing bacteria and their correlation between arsenic (As)-oxidizing bacteria; (2) Sb resistance, mechanism, and influencing factors of Sb-oxidizing bacteria; (3) the rate of Sb(III) oxidation, influencing factors, Sb(III) oxidase, and the oxidation mechanism of Sb-oxidizing bacteria; and (4) application status and prospect of Sb-oxidizing bacteria in environmental remediation. The purpose of this paper is to provide a theoretical basis for understanding the role of Sb-oxidizing bacteria in the process of Sb geochemical cycle, to prevent and control Sb pollution at their source and provide opportunities for bioremediation.

## Screening, Identification, and Diversity of Sb-Oxidizing Bacteria

### Screening and Separation Environment and Separation Conditions of Sb-Oxidizing Bacteria

The microbes that can oxidize Sb(III) to Sb(V) with comparatively low toxicity in the natural environment are called Sb-oxidizing bacteria (Shtangeeva et al., 2011; He et al., 2019). Sb-oxidizing bacteria play a vital role in the biogeochemical cycle of Sb (Filella et al., 2007; He et al., 2019). Compared with As-oxidizing bacteria, although the research on Sb-oxidizing bacteria started relatively late, the screening of Sb-oxidizing bacteria has also achieved considerable advancement since the initial report of Sb-oxidizing bacteria *Stibiobacter senarmontii* in 1974 (Lialikova, 1974). Subsequently, it has been discovered that antimony-oxidizing bacteria are widely distributed in the natural environment. More than 90 strains with antimony oxidation capability have been screened from Sb-contaminated soil or mining sediments in China, South Korea, Japan, Russia, and the United States (Supplementary Table 1). What is worth mentioning is that Li et al. (2013), Shi et al. (2013), Nguyen et al. (2017a), Nguyen and Lee (2015), and other teams have made significant contributions to the field of screening of Sb-oxidizing bacteria. As shown in Supplementary Table 1, more than 80% of Sb-oxidizing bacteria are currently screened from Sb-contaminated soil in China. This result implies that Sb pollution of soil in some mining areas is very serious because of the poor environmental control in the past, and the intrinsic environmental risk from Sb pollution cannot be ignored. On the other hand, it also indicates that Sb pollution in soil has attracted the attention of Chinese scholars and government departments (He et al., 2019). Therefore, with a detailed study in the future, more and more Sb-oxidizing bacteria will be isolated and purified to construct an integrity and diversity Sb-oxidizing bacteria resource database, which lays the foundation for the research and application of Sb-oxidizing bacteria.

Resistance screening that is based on the Sb-contaminated medium is the main method to screen Sb-oxidizing bacteria in the environment. The commonly used bacterial screening media can be divided into two categories: media with a carbon source and media without a carbon source. The media with a carbon source includes chemically defined medium (CDM) (Shi et al., 2013), modified chemically defined medium (M-CDM) (Nguyen et al., 2017a), chemically defined medium CDM-A plates (CDM-A) (Terry et al., 2015), beef extract peptone agar medium (Du et al., 2020), and minimal medium with high carbon conditions or low carbon conditions (LCM/HCM enrichment medium) (Hamamura et al., 2013). According to different metabolic types, Sb-oxidizing bacteria can be divided into chemoautotrophic and heterotrophic. Most of the Sb-oxidizing bacteria are heterotrophic bacteria (Supplementary Table 1), which can be screened and purified using a medium with a carbon source. These bacteria cannot assimilate CO_2_ into cells to synthesize organic molecules to support their growth with oxidizing Sb(III). The energy that is produced by Sb(III) oxidation is mainly used for transport of Sb, DNA damage repair, protein synthesis, and cell movement (Li, 2017; Gu et al., 2019). It may also be partially released in the form of thermal energy (Li, 2017; Gu et al., 2019). The autotrophic Sb-oxidizing bacteria, which can promote their growth by oxidizing Sb(III) and coupling with the stationary CO_2_, can be isolated in a medium without a carbon source. These bacteria include *S. senarmontii* (Lialikova et al., 1976), *Variovorax paradoxus* IDSBO-4 (Terry et al., 2015), *Sulfobacillus* spp., *Leptospirillum* spp., *Ferroplasma* spp., *Sulfobacillus thermotolerans* strain Sb-K, *Sulfobacillus sibiricus* strain Sb-F, and *Sulfobacillus thermosulfidooxidans* strain Sb-S. These studies also suggest that autotrophic Sb-oxidizing bacteria, which can oxidize Sb(III) for energy to survive, probably widely exist in Sb mining areas and Sb-contaminated soils, providing a breakthrough and direction for studying the Sb biogeochemical cycling (Tsaplina et al., 2013).

### Diversity and Phylogenetic Characterization of Sb-Oxidizing Bacteria

**FIGURE 2:**
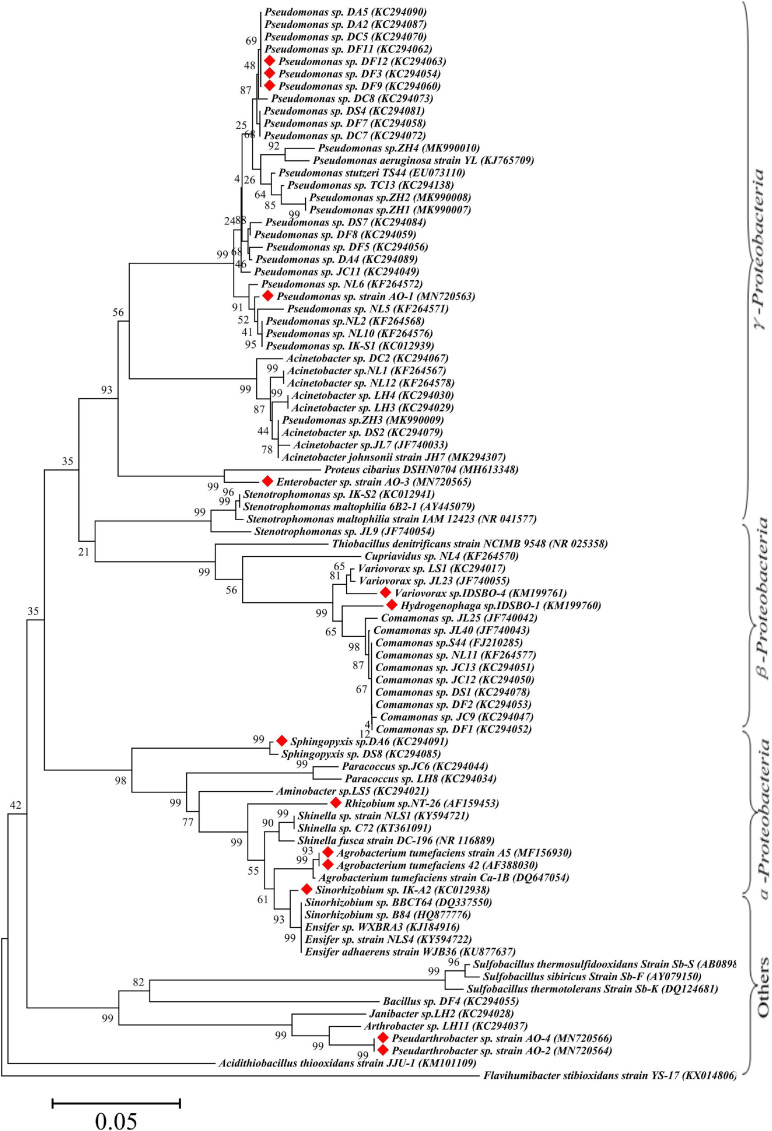
Maximum-likelihood phylogenetic tree of 16S rRNA gene of Sb(III) oxidizers. The red marks represent Sb-oxidizing bacteria with antimony and As oxidation ability.

Bioinformatics analysis is one of the most common and important methods for microbial identification. According to incomplete statistics, about 97 strains with Sb(III) oxidation ability were isolated or discovered so far. The bioinformatics statistical analysis of these strains in phylum, class, order, family, and genus is listed in Supplementary Table 2. On the basis of 16S rRNA gene sequences, a neighbor-joining phylogenetic tree of Sb-oxidizing bacteria (88 strains) was constructed (Figure 2), and their consanguinity was analyzed. As shown in Figure 2, the phylogenetic analysis identified the 88 strains into 26 genera belonging to six major bacterial lineages: α-*proteobacteria* (22.9%), β-*proteobacteria* (17.8%), γ-*proteobacteria* (46.8%), *Actinobacteria (*4.2%), *Firmicutes* (4.2%), and *Bacteroides* (1.0%). These imply that *Proteobacteria*, *Actinomycetes*, *Firmicutes*, and another phylum of microbes are compactly relevant to the biogeochemical cycling of Sb, and Sb-oxidizing bacteria are probably present in these four phyla in which α-, β-, and γ-*proteobacteria* are the most likely phylum, consistent with the conclusions of relevant studies (Wang et al., 2017).

**FIGURE 3:**
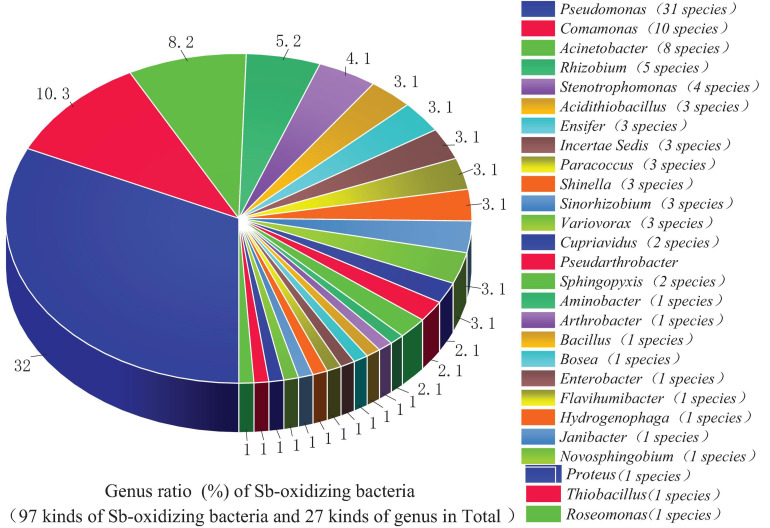
An analysis of Sb-oxidizing bacteria structure at the genus level.

From the perspective of genus level, the reported or isolated Sb-oxidizing bacteria were distributed over 27 genera, including *Pseudomonas* (31.96%), *Comamonas* (10.31%), *Acinetobacter* (9.24%), *Rhizobium* (5.15%), *Stenotrophomona*s (4.12%), *Acidithiobacillus* (3.09%), *Ensifer* (3.09%), *Paracoccus* (3.09%), *Shinella* (3.09%), *Sinorhizobium* (3.09%), *Variovorax* (3.09%), *Cupriavidus* (2.06%), *Pseudarthrobacter* (2.06%), and *Sphingopyxis* (1.03%) (Figure 3). The first five proportions of Sb-oxidizing bacteria in genus, *Pseudomonas*, *Comamonas*, *Acinetobacter*, *Rhizobium*, and *Sphingomonadaceae*, was up to 59.79%. Moreover, the five genera of Sb-oxidizing bacteria are isolated from different Sb mining area, such as Dalong in Guizhou, Ichikawa Antimony Mine, and Xikuangshan (Li et al., 2013; Shi et al., 2013; Terry et al., 2015; Wang et al., 2021), and are commonly known as typical Sb-oxidizing bacteria (Li, 2017). These show that typical Sb-oxidizing bacteria widely exist in Sb-contaminated soils and are the major participants in biogeochemical cycling of Sb (Wang et al., 2018, 2021). Owing to the long-term Sb stress, the typical Sb-oxidizing bacteria are constantly evolving to obtain Sb resistance and the ability to oxidize and play important roles in the biological geochemistry of Sb. However, to the best of our limited knowledge, there are few reports on the typical Sb-oxidizing bacteria. Therefore, in order to comprehend Sb biogeochemical cycling and develop safe and efficient Sb pollution bioremediation technology, it is especially important and urgent to research some related physiological and biochemical characteristics, Sb resistance, and Sb oxidation mechanism of the typical Sb-oxidizing bacteria.

### Correlation Between Sb-Oxidizing Bacteria and As-Oxidizing Bacteria

Due to the similar chemical and toxicological properties of Sb and As (Wen et al., 2018; He et al., 2019), some scholars once believed that the mechanism of microbial oxidation of Sb(III) is exactly the same as that of As(III). In 2007, it was reported that *Agrobacterium tumefaciens* 5A could oxidize As(III) or Sb(III) (Lehr et al., 2007), but the expression of the *aio*A gene, which functions as an As(III) and Sb(III) oxidase, was induced by As(III) but not by Sb(III). Moreover, a disruption of the *aio*A gene caused only 25% decrease of the Sb(III) oxidation. In 2012, some researchers (Xiong et al., 2011; Li et al., 2013) discovered *Comamonas testosteroni* S44 could oxidize Sb(III) but not As(III), while Hamamura et al. (2013) also discovered that *Sinorhizobium* sp. A2 containing the *aioA* gene could only oxidize As(III) but not Sb(III). Terry et al. (2015) found that the autotrophic bacterium *Hydrogenophaga taeniospiralis* IDSBO-1 containing the *aio*A gene could oxidize As(III) but not Sb(III) under aerobic conditions, but it could oxidize Sb(III) using NO_3_^–^ as electron acceptor under anaerobic conditions. These reports showed that microbial oxidation of Sb(III) is catalyzed by a pathway different from the microbial oxidation of As(III) pathway catalyzed by *aio*A. The Sb-oxidizing bacteria and As-oxidizing bacteria that have been reported are distributed in the phyla α-, β-, and γ-*proteobacteria*, but their distribution in small taxa is quite distinguishing. Only a portion of the antimony-oxidizing bacteria can simultaneously oxidize Sb(III) and As(III) (Shi et al., 2013). According to incomplete statistics, there are 14 kinds of strains that oxidize of Sb(III) and As(III) (Figure 3), including α-*proteobacteria* (five strains), γ-*proteobacteria* (five strains), β-*proteobacteria* (two strains), and other (two strains). All of the strains possess AioA enzyme that can catalyze the oxidation reaction of Sb(III) and As(III) (Nguyen and Lee, 2015; Nguyen et al., 2017b). These show that Sb-oxidizing bacteria and As-oxidizing bacteria are not only relevant but also have certain discrepancies. At present, compared to As(III) oxidation, much less information and research method are available for microbial roles in Sb(III) oxidation (Hayat et al., 2017). At the same time, there is a relatively complete As-oxidizing gene and enzyme system that has been discovered with further studies of As-oxidizing bacteria. Therefore, the research methods of As-oxidizing bacteria can be used to research Sb-oxidizing bacteria.

## Antimony Resistance and Mechanism of Sb-Oxidizing Bacteria

### Resistance of Sb-Oxidizing Bacteria

**FIGURE 4:**
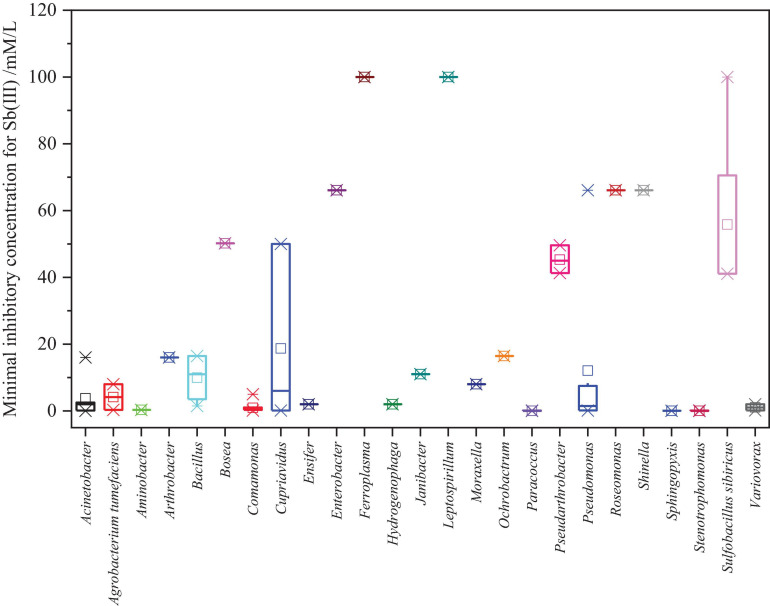
Statistical analysis results of the MIC for Sb(III) of Sb-oxidizing bacteria.

For survival, indigenous microbes have gradually evolved properties and metabolic mechanisms of resistance to potentially toxic metals under long-term metal stress. The minimal inhibitory concentration (MIC), defined as the lowest the concentration of Sb(III) that inhibited bacteria growth, was determined. We summed up the MIC of reported Sb-oxidizing bacteria to Sb(III) and As(III) (Supplementary Table 3 and Figure 4). As shown in Supplementary Table 3, The Sb(III) resistance of Sb-oxidizing bacteria is significantly discrepant with MIC of 0.1–100 mmol/L. It is notable that some indigenous microbes, including *Bosea* sp.AS-1 (50.2 mmol/L) (Lu et al., 2018), *Pseudomonas* sp. AO-1 (66.1 mmol/L), *Enterobacter* sp. AO-3 (66.1 mmol/L), and *Pseudomonas* sp. ZH1 (100 mmol/L) (Hua et al., 2019), which were screened from the serious Sb-polluted soil (16,312–5,000 > mg/kg), exhibited extremely high MIC for Sb(III), and the MIC of the these bacteria outclassed that of non-indigenous bacteria. Therefore, it can be asserted that high Sb-contaminated environment influences Sb-oxidizing bacteria Sb(III) resistance with enhancing their Sb(III) resistance and oxidation ability (Gu et al., 2019). As shown in Figure 4, there were obvious differences between the same genus of Sb-oxidizing bacteria Sb(III) resistance, for instance the Sb(III) resistance of *Pseudomonas* sp. ZH1 (100 mmol/L) (Hua et al., 2019) is about 1,000 times that of *Pseudomonas* sp. DC5 (0.1 mmol/L) (Shi et al., 2013). This implies that the Sb resistance and metabolic mechanism of microbes are multifarious and should be further studied. Furthermore, some Sb-oxidizing bacteria, such as *Sulfobacillus* spp., *Leptospirillum* spp., *Ferroplasma* spp., *S. thermotolerans* strain Sb-K, *S. sibiricus* strain Sb-F, *S. thermosulfidooxidans* strain Sb-S (Tsaplina et al., 2010; Zhuravleva et al., 2011), and *Roseomonas rhizosphaerae* YW11, exhibited higher tolerance to Sb(III) than to As(III). In contrast, some Sb-oxidizing bacteria, such as *Comamonas* sp. S44 (Xiong et al., 2011; Li et al., 2013), *Comamonas* sp. JL25, *Comamonas* sp. JL40, *Variovorax* sp. JL23, *Acinetobacter* sp. JL7, *Stenotrophomonas* sp. JL9, *Bosea* sp. AS-1 (Xiang et al., 2022), and *Pseudomonas stutzeri* TS44, exhibited higher tolerance to As(III) than to Sb(III). There were obvious differences between Sb(III) resistance and As(III) resistance of the same Sb-oxidizing bacteria (Li et al., 2013). The main reason is the different metabolic pathways and resistance mechanisms of microbe to Sb(III) and As(III) and the toxicity of Sb(III) and As(III), but this needs further investigation.

### Sb(III) Resistance Mechanism of Sb-Oxidizing Bacteria

The resistance to Sb(III) of Sb-oxidizing bacteria involves transformations, oxidation–reduction, methylation, and chelation mechanisms (Wysocki et al., 2003; Filella et al., 2007; Li, 2017).

#### Sb Transformations

Antimony is a non-essential element and is toxic to most organisms. Therefore, Sb transformations, including Sb uptake, efflux, and *in vivo* detoxification (Wysocki et al., 2001), play a significant role in the resistance of microbe. One of these, Sb efflux, is a principal and extensive transport mechanism in Sb-oxidizing bacteria.

##### Sb(III) uptake

In 1998, a study by Suzuki et al. (1998) confirmed the glycerol transporter GlpF could transport Sb(III) into *E*scherichia *coli* cell, and homologue Fps1F of GlpF also has the same function in *Saccharomyces cerevisiae* (Sanders et al., 1997; Meng et al., 2004). Subsequently, Wysocki et al. (2001) discovered that when the heavy metal transport gene *fpsl* is disrupted or is down-regulated, the Sb(III) resistance of *S. cerevisiae* is enhanced, indicating *fpsl* gene with the function of Sb(III) uptake. Liu et al. (2002) discovered that AQP1 aquaporin facilitates Sb(III) across *Leishmania* cell membranes, and AQP7 and AQP9 aquaporin also facilitate Sb(III) across mammalian cells. Recently, Gu et al. (2019) discovered the *pst* gene, a transmembrane transporter with Sb(V) uptake, was down-regulated under Sb(III) stress. To the best of our knowledge, there are few studies that relate the specific aquaporin or proteins with the function of Sb(III) uptake.

##### Sb(III) efflux

Sb(III) efflux that can avoid Sb(III) accumulation in cells is one of the primary defense mechanisms of microbes against Sb(III) stress and toxicity. At present, four protein families have been reported to have the function of Sb(III) efflux in bacteria. The first and most important category is ArsB transport protein of ion transport family (Rosen and Borbolla, 1984; Suzuki et al., 1998; Butcher et al., 2000; Rosen, 2002). As Sb(III)/As(III) resistant, *ars* operon may be in bacterial plasmids or chromosomes, and its expression can be induced by Sb(III) and As(III). The *ars* operon can encode some Sb(III)/As(III) resistance genes, including *arsR*, *arsA*, *arsB*, *arsC*, *arsD*, *arsH*, *acr3*, and so on. Among those genes, the *arsB* gene can encode ArsB transport protein, which is a trivalent metal/H^+^ antiporter. When ATP hydrolase ArsA exists, ArsB transport protein can efflux Sb(III)/As(III) with energy produced by hydrolysis of ATP (Xu et al., 1998), and without ArsA, ArsB transport protein can efflux Sb(III)/As(III) using the electron chemical proton gradient generated by itself (Achour et al., 2007; Wang G. et al., 2011). The ArsB transport protein, which regulates Sb(III)/As(III) efflux (Li, 2017), is found widely in Sb-oxidizing bacteria, such as *E. coli* (Carlin et al., 1995), *Pseudomonas aeruginosa* (Ma et al., 1999), *Bacillus subtilis* (Sato and Kobayashi, 1998), *Staphylococcus aureus* (Silver, 1998), and *Acidiphilium multivorum* (Suzuki et al., 1998). More recently, studies have further found that the expression of ArsB is induced by Sb(III) in some microbes that exhibited higher MIC for Sb(III) with up-regulation of ArsB expression, such as *Comamonas* sp. S44 (Li et al., 2013), *A. tumefaciens* 5A (Wang et al., 2015), and *Acinetobacter johnsonii* JH7 (Gu et al., 2020). The second category is Acr3p transport protein of ion transport family (Rosen and Borbolla, 1984; Suzuki et al., 1998; Butcher et al., 2000; Rosen, 2002). The Acr3p protein and its homologous YqcL protein in the As transport family encoded by the *acr* gene cluster (*acr1*, *acr2*, and *acr3*) can substitute the ArsB protein as the Sb(III)/As(III) efflux pump (Achour et al., 2007). The Acr3p transfer protein or *acr3p* gene has been detected in some Sb-oxidizing bacteria, such as *Comamonas* sp. S44 (Li et al., 2013), *A. tumefaciens* 5A (Wang et al., 2015), *Bosea* sp. AS-1 (Lu et al., 2018), and *R. rhizosphaerae* strain YW11 (Sun et al., 2020). Li et al. (2017) found that disruption of *acr3* gene would reduce the Sb(III) resistance of *A. tumefaciens* 5A. This study directly confirmed that Acr3p transport protein was associated with Sb(III) resistance (Bobrowicz et al., 1997; Maciaszczyk-Dziubinska et al., 2011; Kang et al., 2014). With the helping of real-time PCR technology, Luo et al. (2014) further found that the abundance of ArsB and Acr3 in soil was positively correlated with the concentration of As and Sb, implying that Sb(III)/As(III) could induce the expression of *arsB* and *acr3* resistance genes and increase Sb(III)/As(III) resistance in most bacteria. Therefore, ArsB and Acr3p transport proteins are universal Sb(III) efflux pump. The third category is the ABC transporter family in eukaryotes (Ghosh et al., 1999; Manzano et al., 2013). The ABC transporter YCF1 in *S. cerevisiae* increases the Sb(III) resistance by accumulating Sb–glutathione (GSH) compounds in the vacuole. The fourth category contains transport proteins or enzymes, including glycerol channel protein GlpF, Fpslp, CzcA, CopA, and CopB enzymes. GlpF can transport Sb(III) into cells in *E. coli*, and Fpslp and Acr3 have the function of Sb(III) efflux in *S. cerevisiae* (Bobrowicz and Uaszewski, 1998; Wysocki et al., 2001). Recently, Gu et al. (2019) found that the regulation of CzcA, CopA, and CopB enzymes, which were defined as functional Sb(III) efflux, was up-regulated under Sb(III) stress. To summarize, due to the diversity and complexity of proteins or enzymes, the ones required for Sb(III) efflux are still unknown and need further investigation.

##### Sb(V) transformation mechanism

At present, few reports exist specifically reporting the mechanism of Sb(V) transformation by microbes. Moreover, there are insignificant differences between Sb(V) transformation and Sb(III) transformation (Christian et al., 2003), and there were two probable microbial Sb(V) transformation mechanisms. The first, similar to As(V), Sb(V) is transported by immediate (specific) channels or transport family proteins, such as the phosphate transport system Pit or Pst (Gu et al., 2020). The second, Sb(V) is first reduced to Sb(III), followed by Sb(III) uptake or efflux (Xu et al., 1998; Martin et al., 2001). In addition, Gu et al. (2020) considered that there may be more kinds of Sb(V) transformation mechanisms in microbes, but it needs to be confirmed.

#### Antimony Oxidation–Reduction

##### Oxidation

The conversion between Sb(III) and Sb(V) is restricted and affected by abiotic and biological factors. Microbial oxidation that converts the toxic Sb(III) to less-toxic Sb(V) improves the microbial tolerance to Sb (Leuz and Johnson, 2005) and the adaptability to natural environment. Moreover, some chemoautotroph Sb-oxidizing bacteria, such as *S. senarmontii* (Lialikova, 1974; Lialikova et al., 1976), can utilize the energy generated from Sb(III) oxidation reaction to maintain normal growth. As shown in Supplementary Table 3, the more than 90 indigenous Sb-oxidizing bacteria exhibited higher MIC for Sb(III) than the ordinary bacteria. The results implied that Sb resistance of Sb-oxidizing bacteria is associated with their biological oxidative ability and has nonlinear relationships (Li et al., 2013). The reason maybe is that the Sb(III) resistance of Sb-oxidizing bacteria is determined by Sb transformations (uptake and efflux), redox, and methylation, and the role of oxidation in the Sb(III) resistance of different Sb-oxidizing bacteria is distinct.

##### Reduction

Under anaerobic conditions, some microbes can reduce Sb(V) to Sb(III). Kantin (1983) first reported that the macroalgae *Sargassum* sp. can reduce Sb(V) to Sb(III) in seawater. In 2014, researchers (Shaked-Mishan et al., 2001; Ferreira et al., 2003; Zhou et al., 2004; Hansen et al., 2011) discovered some reducing substances, such as As reductase Acr2 or glutathione, also serve the same function. Recently, some scholars (Liu et al., 2018; Zhu et al., 2018) discovered various Sb-reducing bacteria and found that some of them have a dissimilation respiration capacity during the reduction of Sb(V). This means that there is a metabolic mechanism of Sb(V) as the energy source, which is prominently associated with its Sb resistance (Ca and Jt, 2014; Kulp et al., 2015). In addition, Sb-reducing bacteria, such as *Bacillus* sp. MLFW-2 (Zhu et al., 2018) and *Sinorhizobium* sp. JUK-1 (Nguyen and Lee, 2014; Rai et al., 2018), will produce biologically generated secondary minerals during the reduction of Sb(V) to Sb(III) (Zhu et al., 2018), which lays the foundation for application to the extraction of low-grade antimony ores and microbial remediation technology (Zhang et al., 2019).

#### Antimony Methylation

Antimony biomethylation refers to catalytic transfer of the methyl groups of active methyl compounds to Sb and its compounds in the biological metabolic process. The three forms of Sb methylation are monomethylated Sb CH_3_SbH_2_ (MMSb), dimethylated Sb (CH_3_)_2_SbH (DMSb), and trimethylated Sb (CH_3_)_3_Sb (TMSb) (Gürleyük et al., 1997). Previous studies found that Sb methylation widely occurred in fungi, archaea, and bacteria (Zhang et al., 2021b). Jenkins et al. (1998) discovered filamentous fungi *Scopulariopsis brevicaulis* and *Phaeolus schweinitzii* could convert inorganic potassium Sb tartrate into TMSb under aerobic conditions. Subsequently, Michalke et al. (2000) also reported that the formation of TMSb occurred under anaerobic conditions in *Clostridium collagenovorans* and *Desulfovibrio vulgaris*. *Methanobacterium formicicum* could convert inorganic Sb compounds into MMSb or DMSb. Gürleyük et al. (1997) discovered that the aerobic bacteria *Pseudomonas fluorescens* could produce trimethyl antimony bromide. Smith et al. (2002) reported that *Cryptococcus humicolus* could convert Sb(V) into TMSb. Jenkins et al. (2002) found that *Flavobacterium* sp. also has a methylation function. Meyer et al. (2007) discovered that anaerobic cultured Gram-positive bacteria *Clostridium glycolicum* could convert inorganic Sb into volatile methyl compounds DMSb and TMSb. In addition, both *C. glycolicum* AS and *Flavobacterium* sp. can convert inorganic Sb into DMSb and TMSb (Jenkins et al., 2002; Meyer et al., 2007). Due to similar chemical properties, Sb methylation and As methylation are correlated with the similar biological process and mechanism. However, As biomethylation reactions occur more widely than those of Sb (Andrewes et al., 2000). Moreover, trace As can promote Sb methylation reaction in the environment, but the presence of Sb can inhibit As methylation reaction. Several studies (Andrewes et al., 2000; Wehmeier and Feldmann, 2005) had shown that As methylase and S-methionine methyltransferase play a crucial role in the process of Sb(III) methylation, and GSH and methylcobalamin may be involved in the process of Sb(V) methylation. However, the genes or enzymes required for Sb methylation are still unknown. In addition, it is generally deemed that Sb methylation was more likely to occur in Sb(III) than in Sb(V) compounds (Andrewes et al., 2000; Filella, 2010). In summary, Sb methylation has been regarded as a detoxification process, which is prominently associated with Sb resistance (Jenkins et al., 2002), but generated more migratory organic antimony, resulting in global Sb migration and pollution (Herath et al., 2017). Overall, Sb biomethylation plays a key role in the biogeochemical cycle of Sb and is closely related with public health, but the genes or enzymes required for Sb biomethylation are still unknown and need further study.

#### Environmental Factors

In order to enhance tolerance to Sb and maintain survival, Sb-oxidizing bacteria can synthesize more cell wall and peroxide under exposure to Sb(III). Gu et al. (2020) found that the expression of outer membrane protein and its assembly factor (YgfL, BamE, lipoprotein-34, MltD, Ddl, OMPP1, LpxB, LptE, LptD, and 28-kDa outer membrane protein) of *A. johnsonii* JH7 was up-regulated under Sb(III) stress and thus would synthesize more extracellular polymer (EPS), which could adsorb Sb(III) and reduce Sb uptake. Several studies (Wang et al., 2015; Li, 2017; Gu et al., 2020) have shown that the contents or activities of GST, CAT, and GPx (peroxidase) increased in microbes under Sb(III) stress, which promotes the conversion from the toxic Sb(III) to less-toxic Sb(V), resulting in improving the microbial tolerance to Sb (Leuz and Johnson, 2005). Under Sb(III) stress, the expression of ribosomal subunits with translation and protein metabolism; dihydrolipoyl dehydrogenase and phosphoenolpyruvate carboxylase related to energy metabolism; proteins associated with TCA cycle; and oxidative phosphorylation genes is up-regulated. These findings indicated that microbes took more energy under Sb(III) stress, than under a normal condition (Li, 2017), and increased energy requirements principally were used for Sb transformations, DNA damage repair, protein synthesis, and cell movement (Li, 2017). Environmental factors will affect the growth and reproduction of bacteria, but how these affect bacterial Sb resistance is still unknown. Based on the analysis of 125 Sb-resistant strains (36 Sb-oxidizing bacteria), Shi et al. (2013) found that the concentration of Sb and copper in the soil was the signification factor affecting bacterial Sb resistance, but other environmental factors (O-M, S, N, P, NO_3_^–^, Fe, As, and pH in soil) did not reach significant correlation with the MIC for Sb(III) (*p* > 0.05). Subsequently, Luo et al. (2014) also found that the abundance of ArsB and Acr3, as well as the MIC for Sb(III), was positively correlated with the As and Sb concentrations in the soil. However, Lu et al. (2018) found that the As resistance of *Bosea* sp. AS-1 was uncorrelated with the concentration of As in the environment. To the best our knowledge, there are few literatures that report the related environmental factors affecting the Sb resistance of Sb-oxidizing bacteria. Therefore, how environmental factors affect the Sb resistance of Sb-oxidizing bacteria is still unclear and needs further research.

### Sb Resistance Mechanism of Sb-Oxidizing Bacteria

**FIGURE 5:**
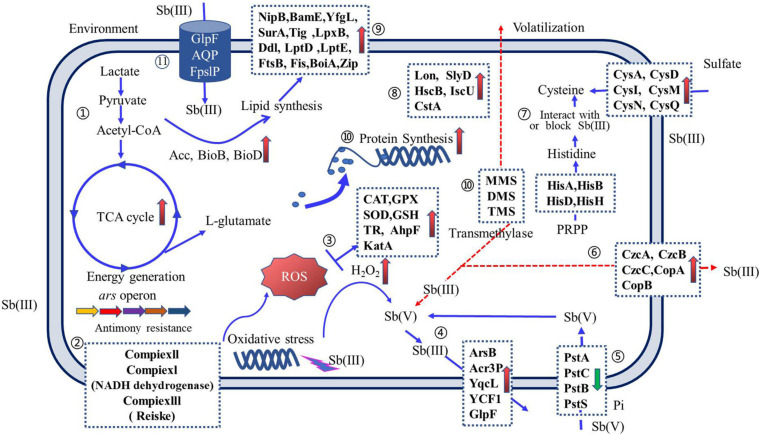
An overview of Sb(III) resistance regulation of Sb-oxidizing bacteria. Arrows on solid blue lines (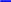) indicate the function of proteins that have been reported and validated; arrows on dashed red lines (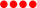) indicate proposed protein functions and have not been reported elsewhere or require further validation. Up-regulated proteins (upward pink arrows) and down-regulated proteins (downward green arrow) are indicated. Pathways include the following: ➀ carbon metabolism; ➁ oxidative phosphorylation; ➂ reactive oxygen species (ROS) scavenging; ➃ Sb efflux; ➄ phosphate transporters; ➅ metallophores; ➆ amino acid synthesis; ➇ anti-stress proteins; ➈ cell wall formation; ➉ ribosomal and peptide syntheses; and (11) Sb(III) uptake (for interpretation of the references to color in this figure legend, the reader is referred to the web version of this article).

The Sb(III) resistance of Sb-oxidizing bacteria is mainly determined by the regulation system of intracellular Sb concentration, Sb valence transformation system with detoxification (Lialikova, 1974; Terry et al., 2015), energy metabolism system, cell wall formation system, and peroxide generation system (Liu et al., 2015; Wang et al., 2016; Li, 2017). Therefore, the Sb(III) resistance mechanism of Sb-oxidizing bacteria is a complex system (Li et al., 2015; Gu et al., 2020), involving more than a dozen biochemical reactions, such as Sb transformations, oxidation–reduction, methylation, and chelation mechanisms (Wysocki et al., 2003; Filella et al., 2007; Li, 2017; as shown in Figure 5).

## The Performance and Molecular Mechanism of Sb-Oxidizing Bacteria

### Sb-Oxidizing Bacteria Oxidation Rate for Sb(III)

Microbial Sb(III) oxidation, which transforms Sb(III) to Sb(V), could be considered a means of detoxification because the toxicity of Sb(III) is orders of magnitude more than that of Sb(V) (He et al., 2019). Moreover, Sb(V) is very stable with high solubility and mobility in solution. So, microbial Sb(III) oxidation plays a crucial role in Sb speciation, mobility, bioavailability, and fate in the aquatic environment (Thanabalasingam and Pickering, 1990; Leuz et al., 2006a; Li, 2017). Asta et al. (2012) reported that the abiotic dissolution and oxidation of Sb(III) from low-grade ore, such as Sb_2_S_3_, FeSb_2_S_4_, and Sb_2_O_3_, are extremely slow at a neutral condition with a half-life of 170 days. The oxidation rate of Sb(III) was significantly promoted by redox changes in the soil (Hockmann et al., 2014). Studies (Blackmore et al., 2018) further found that the release rate of Sb(III) from antimony ore waste rock was significantly enhanced under the microbial mediation. It can be seen that this mediation can vastly accelerate the dissolution and oxidation rate of Sb(III) from Sb-bearing minerals and rocks. So, microbial Sb(III) oxidation is one of the most important driving forces for the formation, migration, transformation, and fate of Sb pollution in the mining area (Tsaplina et al., 2010). It is thus of great significance to understand the Sb(III) oxidation mechanism, as these processes will greatly enhance our understanding of the Sb biogeochemical cycle and its environmental impacts (Loni et al., 2020).

The oxidation rate of Sb(III) is a vital index to evaluate the oxidation performance of Sb-oxidizing bacteria and also reflect the contribution of Sb-oxidizing bacteria in Sb biogeochemical cycling. So, determining the oxidation rate of Sb(III) has been of considerable interest. The results of the oxidation rate of Sb(III) are presented in Table 1. The maximum oxidation rate (*V*_max_) of *Paracoccus versutus* XT0.6 is up to 902.88 μM/day under aerobic conditions (Loni et al., 2020), then followed by *Ensifer* sp. strain NLS4 (460.8 μM/day) and *Shinella* sp. strain NLS1 (403.2 μM/day) (Nguyen et al., 2017a). Due to the uncertainty and difficulty in determining the maximum oxidation rate of Sb(III), the average oxidation rate of Sb(III) (*V*_avg_) is conventionally determined. The *V*_avg_ of different Sb-oxidizing bacteria is 0.10–300 μM/day (Table 1), and the oxidation performance has significant discrepancies (Li et al., 2013; Nguyen et al., 2017a). Moreover, the *V*_avg_ for different Sb-oxidizing bacteria varies widely. The *V*_avg_ of *Pseudomonas* sp. AO-1, *Pseudomonas* sp. NL6 were 48 and 1.72 μM/day, respectively. In addition, some Sb-oxidizing bacteria, such as *Shinella* sp. strain NLS1 (66.7 μM/day) (Nguyen et al., 2017a), *H. taeniospiralis* strain IDSBO-1 (55.56 μM/day), and *P. versutus* XT0.6 (100 μM/day) (Loni et al., 2020), exhibited Sb(III) oxidation ability with nitrate as the electron acceptor under anaerobic conditions. These results indicated that some Sb-oxidizing bacteria have disparate metabolic pathways and mechanisms for Sb(III) under aerobic and anaerobic conditions, but further study is needed.

**TABLE 1:** Comparison of oxidation rate of Sb(III) for Sb-oxidizing bacteria.

Name of the Sb-oxidizing bacteria	Initial form of Sb(III)	Initial concentration of Sb(III)	*V*_max_ or Sb(III) oxidation rate (*V*_avg_)	Culture condition	Carbon source	Analytical method	References
*Cupriavidus* sp. NL4	C_8_H_4_K_2_O_12_Sb_2_	100 μM	4.74 μM/day	Aerobic	Lactate	Nonpolar SPE cartridges + ICP-MS	Nguyen and Lee, 2015
*Comamonas* sp. NL11	C_8_H_4_K_2_O_12_Sb_2_	100 μM	1.72 μM/day	Aerobic	Lactate		
*Acinetobacter* sp. NL1	C_8_H_4_K_2_O_12_Sb_2_	100 μM	0.96 μM/day	Aerobic	Lactate		
*Acinetobacter* sp. NL12	C_8_H_4_K_2_O_12_Sb_2_	100 μM	2.33 μM/day	Aerobic	Lactate		
*Pseudomonas* sp. NL2	C_8_H_4_K_2_O_12_Sb_2_	100 μM	4.24 μM/day	Aerobic	Lactate		
*Pseudomonas* sp. NL5	C_8_H_4_K_2_O_12_Sb_2_	100 μM	4.40 μM/day	Aerobic	Lactate		
*Pseudomonas* sp. NL6	C_8_H_4_K_2_O_12_Sb_2_	100 μM	1.72 μM/day	Aerobic	Lactate		
*Pseudomonas* sp. NL10	C_8_H_4_K_2_O_12_Sb_2_	100 μM	3.95 μM/day	Aerobic	Lactate		
*Pseudomonas* sp. IK-S1	C_8_H_4_K_2_O_12_Sb_2_	100 μM	2.57 μM/day	Aerobic	Lactate	ICP-OES	Hamamura et al., 2013
*Stenotrophomonas* sp. IK-S2	C_8_H_4_K_2_O_12_Sb_2_	100 μM	2.01 μM/day	Aerobic	Lactate	ICP-OES	Hamamura et al., 2013
*Acinetobacter* sp. JL7	C_8_H_4_K_2_O_12_Sb	50 μM	1.25 μM/day	Aerobic	Lactate	HPLC-HG-AFS	Li et al., 2013
*Comamonas* sp. JL25	C_8_H_4_K_2_O_12_Sb_2_	50 μM	<0.1 μM/day	Aerobic	Lactate	HPLC-HG-AFS	Li et al., 2013
*Comamonas* sp. JL40	C_8_H_4_K_2_O_12_Sb_2_	50 μM	81 μM/day/10	Aerobic	Lactate	HPLC-HG-AFS	Li et al., 2013
*Comamonas* sp. S44	C_8_H_4_K_2_O_12_Sb_2_	50 μM	16.67 μM/day	Aerobic	Lactate	HPLC-HG-AFS	Li et al., 2013
*Stenotrophomonas* sp. JL9	C_8_H_4_K_2_O_12_Sb_2_	50 μM	1.89 μM/day	Aerobic	Lactate	HPLC-HG-AFS	Li et al., 2013
*Variovorax* sp. JL23	C_8_H_4_K_2_O_12_Sb_2_	50 μM	6.67 μM/day	Aerobic	Lactate	HPLC-HG-AFS	Li et al., 2013
*Arthrobacter* sp. LH11	C_8_H_4_K_2_O_12_Sb_2_	50 μM	3.3 μM/day	Aerobic	Lactate	HPLC-HG-AFS	Li et al., 2013
*Acinetobacter* sp. LH3	C_8_H_4_K_2_O_12_Sb_2_	10 μM	3.33 μM/day	Aerobic	Lactate	HPLC-HG-AFS	Li et al., 2013
*Sphingopyxis* sp. DA6	C_8_H_4_K_2_O_12_Sb_2_	10 μM	3.53 μM/day	Aerobic	Lactate	HPLC-HG-AFS	Li et al., 2013
*Sphingopyxis* sp. DS8	C_8_H_4_K_2_O_12_Sb_2_	10 μM	3.53 μM/day	Aerobic	Lactate	HPLC-HG-AFS	Li et al., 2013
*Cupriavidus* sp. S1	C_8_H_4_K_2_O_12_Sb_2_	100 μM	4.17 μM/day	Aerobic	Lactate	ICP-OES	Shi et al., 2013
*Moraxella* sp. S2	C_8_H_4_K_2_O_12_Sb_2_	100 μM	<1.53 μM/day	Aerobic	Lactate	ICP-OES	Shi et al., 2013
*Bacillus* sp. S3	C_8_H_4_K_2_O_12_Sb_2_	100 μM	50 μM/day	Aerobic	Lactate	ICP-OES	Shi et al., 2013
*Bosea* sp. AS-1	C_8_H_4_K_2_O_12_Sb_2_	2 mM	333.3 μM/day*	Aerobic	Lactate	HPLC-HG-AFS	Shi et al., 2013
*Variovorax paradoxus* strain IDSBO-4	C_8_H_4_K_2_O_12_Sb_2_	1,000 μM	80 μM/day	Aerobic	Without yeast	HPLC-HG-ICS	Shi et al., 2013
*Hydrogenophaga taeniospiralis* strain IDSBO-1	C_8_H_4_K_2_O_12_Sb_2_	1,000 μM	55.56 μM/day	Anaerobic	Without yeast	HPLC-HG-ICS	Shi et al., 2013
*Paracoccus versutus* XT0.6	C_8_H_4_K_2_O_12_Sb_2_	300 μM	300 μM/day 902.88 μM/day*	Aerobic	Lactate	HPLC-HG-AFS	Loni et al., 2020
	C_8_H_4_K_2_O_12_Sb_2_	300 μM	100 μM/day	Anaerobic	Lactate	HPLC-HG-AFS	Loni et al., 2020
	(30–97%) Sb_2_S_3_	–	13.7–38.0 μM/day	Anaerobic	Lactate	HPLC-HG-AFS	Loni et al., 2020
*Shinella* sp. strain NLS1	C_8_H_4_K_2_O_12_Sb_2_	500 μM	403.2 μM/day*	Aerobic	Yeast or without yeast	LAM-ICP-MS	Nguyen et al., 2017a
*Shinella* sp. strain NLS1	C_8_H_4_K_2_O_12_Sb_2_	200 μM	0 μM/day	Anaerobic	Yeast	LAM-ICP-MS	Nguyen et al., 2017a
*Ensifer* sp. strain NLS4	C_8_H_4_K_2_O_12_Sb_2_	500 μM	460.8 μM/day*	Aerobic	Yeast	LAM-ICP-MS	Nguyen et al., 2017a
*Ensifer* sp. strain NLS4	C_8_H_4_K_2_O_12_Sb_2_	500 μM	66.7 μM/day	Aerobic	Yeast or without yeast	LAM-ICP-MS	Nguyen et al., 2017a
*Agrobacterium tumefaciens* GW4	C_8_H_4_K_2_O_12_Sb_2_	50 μM	150 ± 7 nmol min^–1^ mg^–1^*	Anaerobic	lactate	HPLC-HG-AFS	Li et al., 2015
*Agrobacterium tumefaciens* sp. 5A	Sb(CH_3_)_3_Cl_2_	50 μM	2.86 μM/day	Aerobic	Lactate	HPLC-HG-AFS	Lehr et al., 2007
*Rhizobium* strain NT-26	C_8_H_4_K_2_O_12_Sb_2_	164 μM	7.3 μM/day 18.4 ± 1.2 nmol min^–1^ mg^–1^*	Aerobic	Lactate	HPLC-HG-AFS	Wang et al., 2015
*Sulfobacillus thermotolerans* strain Sb-K	Sulfide minerals	Contain Sb_S_, 10.8%	2.54 μM/day	Aerobic	Yeast or glucose	**–**	Tsaplina et al., 2010; Zhuravleva et al., 2011
*Sulfobacillus sibiricus* strain Sb-F	Sulfide minerals	Contain Sb_S_, 10.8%	2.26 μM/day	Aerobic	Yeast or glucose	**–**	Tsaplina et al., 2010; Zhuravleva et al., 2011
*Sulfobacillus thermosulfidooxidans* strain Sb-S	Sulfide minerals	Contain Sb_S_, 10.8%	2.16 μM/day	Aerobic	Glucose	**–**	Tsaplina et al., 2010; Zhuravleva et al., 2011
*Pseudomonas stutzeri* TS44	C_8_H_4_K_2_O_12_Sb_2_	200 μM	48 μM/day	Aerobic	Lactate	HPLC-HG-AFS	Wang et al., 2016
*Stenotrophomonas maltophilia* str. IAM 12423	C_8_H_4_K_2_O_12_Sb_2_	500 μM	1.14 μM/day	Aerobic	Lactate	ICP-OES	Gu et al., 2019
*Pseudomonas* sp. AO-1	C_8_H_4_K_2_O_12_Sb_2_	500 μM	48 μM/day	Aerobic	Lactate	ICP-OES	Author
*Pseudarthrobacter* sp. AO-2	C_8_H_4_K_2_O_12_Sb_2_	500 μM	95.63 μM/day	Aerobic	Lactate	ICP-OES	
*Enterobacter* sp. AO-3	C_8_H_4_K_2_O_12_Sb_2_	500 μM	54.3 μM/day	Aerobic	Lactate	ICP-OES	
*Pseudarthrobacter* sp. AO-4	C_8_H_4_K_2_O_12_Sb_2_	500 μM	73.14 μM/day	Aerobic	Lactate	ICP-OES	
Microbial oxidation (non-purebred)	Sb2O3	0.2–21 μM	0.96–1.20 μM/day	Aerobic	AMD-inoculated synthetic acid medium	HG-AAS	Asta et al., 2012
*Pseudomonas* sp. ZH1	C_8_H_4_K_2_O_12_Sb_2_	100 μM	6.0 μM/day	Aerobic	Lactate	ICP-OES	Hua et al., 2019
*Pseudomonas* sp. ZH2	C_8_H_4_K_2_O_12_Sb_2_	100 μM	6.0 μM/day	Aerobic	Lactate	ICP-OES	Hua et al., 2019
*Pseudomonas* sp.ZH3	C_8_H_4_K_2_O_12_Sb_2_	100 μM	7.0 μM/day	Aerobic	Lactate	ICP-OES	Hua et al., 2019
*Pseudomonas* sp. ZH4	C_8_H_4_K_2_O_12_Sb_2_	100 μM	9.5 μM/day	Aerobic	Lactate	ICP-OES	Hua et al., 2019
*Cupriavidus* strain Dmm 5T-5-1	C_8_H_4_K_2_O_12_Sb_2_	100 μM	6.0 μM/day	Aerobic	Lactate	ICP-OES	Hua et al., 2019
*Acinetobacter johnsonii* JH7	C_8_H_4_K_2_O_12_Sb_2_	824.4 μM	0.077 μM/day	Aerobic	Yeast + lactate	ICP-OES	Gu et al., 2020
*Roseomonas rhizosphaerae* strain YW11	C_8_H_4_K_2_O_12_Sb_2_	20 μM	4.95 μM/day	Aerobic	Yeast	HPLC-HG-AFS	Sun et al., 2020
*Roseomonas cervicalis* ATCC49957	C_8_H_4_K_2_O_12_Sb_2_	20 μM	3.64 μM/day	Aerobic	Yeast	HPLC-HG-AFS	Sun et al., 2020
*Roseomonas ludipueritiae* DSM14915	C_8_H_4_K_2_O_12_Sb_2_	20 μM	3.80 μM/day	Aerobic	Yeast	HPLC-HG-AFS	Sun et al., 2020

*The maximum oxidation rate of Sb(III), and the rest are the average oxidation rate; the papers of the “Author’s research group” are being published and have not yet been published.

*SPE, solid phase extraction; LAM, laser-ablation microprobe; ICP, inductively coupled plasma; MS, mass spectrometry; HG, hydride generation; AAS, atomic absorption spectroscopy; OES, optical emission spectrometry; HPLC, high-performance liquid chromatography; AFS, atomic fluorescence spectrometry; ICS, image correlation spectroscopy.*

### Influencing Rate of Sb(III) Oxidation by Sb-Oxidizing Bacteria

The microbial oxidation rate of Sb(III) is closely associated with their living environment (abiotic factor) and genetic characteristic (Li et al., 2021). Abiotic factors, such as Sb form/solid phase, temperature, pH, type of carbon source, electron acceptor [dissolved oxygen (DO), nitrate], redox potential (Eh), and presence of iron/manganese (hydr)oxides, play a part in microbial oxidation of Sb(III). Although microbial oxidation of Sb(III) was already reported, the underlying abiotic factors are largely unexplored and not elucidated. Bacterial genetic characteristics mainly include the diversity and expression abundance of Sb(III)-oxidizing genes or enzymes (Li et al., 2013; Luo et al., 2014).

#### Abiotic Factors

##### Sb formation

The existence of Sb will affect the microbial oxidation rate of Sb(III). As shown in Table 1, Sb(III) in the form of C_8_H_4_K_2_O_12_Sb_2_ was often used to determine microbial oxidation rate in the laboratory. Loni et al. (2020) found that the oxidation rate of Sb(III) in the form of C_8_H_4_K_2_O_12_Sb_2_ by *P. versutus* XT0.6 is much faster than that of Sb(III) in the form of Sb_2_S_3_. Asta et al. (2012) also reported that the oxidation rate of Sb_2_O_3_ by mixed microbial population is only 0.96–1.20 μM/day, and it is far less than that determined for C_8_H_4_K_2_O_12_Sb_2_. In nature, antimony mainly exists in the form of sulfides and oxides, such as antimonite (Sb_2_S_3_), valentinite (Sb_2_O_3_), senarmontite (Sb_2_O_3_), cervantite (Sb^3+^Sb^5+^O_4_), kermesite (Sb_2_S_2_O), stibiconite [Sb_3_O_6_(OH)], and so on (Herath et al., 2017; Loni et al., 2020). However, little research had been published to discuss the oxidation rate of these minerals by Sb-oxidizing bacteria (Loni et al., 2020). So, it is necessary to hand insight into the geochemical process of dissolution and oxidation of Sb(III) from these minerals, as these processes may greatly enhance our understanding about the process of Sb pollution formation from Sb-bearing minerals and rocks and put forward control countermeasures.

##### Temperature

Temperature may affect the growth of Sb-oxidizing bacteria and then affect their oxidation rate. At present, the microbial oxidation rate of Sb(III) was determined at the temperature of 15–30°C in the laboratory. Up to now, only a few papers had been published to discuss temperature influences on the oxidation rate of Sb(III). Lu et al. (2018) found that the growth and oxidation rate of Sb(III) for *Bosea* sp. AS-1 was not significantly affected between 25 and 40°C. At the same time, Gu et al. (2020) also reported that the optimum oxidation temperature for *A. johnsonii* JH7 was 30°C, and the oxidation rate of Sb(III) was not significantly affected between 10 and 40°C. Overall, the appropriate growth temperature has a little effect on the microbial oxidation rate of Sb(III).

##### Carbon source

The carbon source is an essential substance for the growth of microbes. But now, there is rather a dispute about how carbon source affect the oxidation rate of Sb(III) for Sb-oxidizing bacteria. Some researchers believe that the microbial oxidation rate of Sb(III) was not affected by carbon source. For example, the oxidation rate of Sb(III) by *Shinella* sp. strain NLS1 and *Ensifer* sp. strain NLS4 was not substantially affected under the presence or absence of yeast in the CDM (Nguyen et al., 2017a), and studies comparing changes in oxidation rate of Sb(III) for *A. johnsonii* JH7 among different carbon sources [CDN-A medium, minimal salt medium (MSM), and Luria-Bertani medium (LBM)] have yielded similar results with Gu et al. (2019). Moreover, Hamamura et al. (2013) reported that the oxidation rate of Sb(III) by *P. stutzeri* TS44 was not significantly different on culture conditions of high-carbon source (containing 10 mmol sodium acetate) or low-carbon source (containing 0.002% yeast). On the other hand, it is considered that the microbial oxidation rate of Sb(III) is affected by carbon source. Hamamura et al. (2013) also reported that the oxidation rate of Sb(III) by *Stenotrophomonas maltophilia* str. IAM 12423 decreased when the carbon source acetate was replaced by yeast carbon source. Using yeast extract as the sole carbon source, the oxidation rate of As(III) for *Bosea* sp. AS-1 is faster than the oxidation rate of Sb(III) (Lu et al., 2018). In contrast, using sodium acetate as the sole carbon source, the oxidation rate of Sb(III) is greater than that of As(III) (Lu et al., 2018). Moreover, yeast extract was beneficial to the oxidation of Sb(III) for *Bosea* sp. AS-1 than sodium acetate (Lu et al., 2018). In addition, our group has also found that glucose carbon source was more conducive to adsorb and oxidize Sb(III) for *Rhodotorula mucilaginosa* than sucrose, galactose, or maltose in aqueous solution (Jin et al., 2020). These studies show that the oxidation rate of Sb(III) for Sb-oxidizing bacteria was different under different carbon sources and culture conditions, and they also imply that Sb-oxidizing bacteria might have a variety of antimony oxidation genes and different Sb metabolic pathways, which can be selected according to the carbon source. However, the relationship between the carbon source and the antimony metabolic pathway needs to be further studied.

##### Electron acceptor

Both DO and nitrate are common electron acceptors (Zhang et al., 2021a). The types of electron acceptors have significant effects on the biological process, cell component, and molecular function of microbes. Previous studies showed that most species of Sb-oxidizing bacteria can oxidize Sb(III) using DO as electron acceptor, and only a few of them can oxidize Sb(III) using nitrate as electron acceptor. The microbial oxidation rate of Sb(III) is obviously different using DO or nitrate as electron acceptor; for instance, the oxidation rate of Sb(III) for *P. versutus* XT0.6 was up to 300 μM/day using DO as electron acceptor while it was only 100 μM/day using nitrate as electron acceptor (Loni et al., 2020). Nguyen et al. (2017a) also reported that the oxidation rate of Sb(III) for the *Ensifer* sp. strain NLS4 was 460.8 and 66.7 μM/day under aerobic and anaerobic conditions, respectively. The *V*_max_ for *Shinella* sp. strain NLS1 was 403.2 μM/day using DO as electron acceptor, but it cannot oxidize Sb(III) using nitrate as electron acceptor (Nguyen et al., 2017a). Similar experimental results were obtained for *V. paradoxus* strain IDSBO-4 by Terry et al. (2015). On the contrary, *H. taeniospiralis* strain IDSBO-1 can oxidize Sb(III) only in the presence of nitrate (55.56 μM/day). These studies indicate that Sb-oxidizing bacteria have different Sb metabolism pathways and oxidation mechanisms using DO or nitrate as electron acceptor. Simultaneously, the oxidation rate of Sb(III) for most species of Sb-oxidizing bacteria is faster under aerobic than that under anaerobic conditions. Therefore, Sb-bearing minerals and rock surfaces are prone to oxidation reaction, which are also the primary sites of Sb pollution formation, migration, and transformation. This is of great significance to the prevention and control of Sb pollution and the development and application of microbial remediation technology.

##### pH

pH is a critical factor affecting the microbial oxidation rate of Sb(III) (Herath et al., 2017; Loni et al., 2020). The oxidation rate of Sb(III) using DO as electron acceptor is very slow in the natural water and is affected by pH and water composition (Leuz and Johnson, 2005). A previous study reported that Sb(III) was scarcely oxidized for 35 days at pH < 7.0 in water, but approximately 30% of Sb(III) was oxidized to Sb(V) at pH = 9.0 (Xi et al., 2013). Sb(III) dissolution and oxidation from minerals is also a pH-dependent process (Hu et al., 2015). As such, the oxidation rate of Sb(III) from Sb sulfide minerals increases with increasing pH, but the oxidation rate of Sb(III) from Sb oxide minerals increases with decreasing pH. Some previous studies deemed that pH affects the microbial oxidation rate of Sb(III) in four different ways (Herath et al., 2017; Loni et al., 2020). Firstly, neutral and weakly alkaline conditions are conducive to the growth and reproduction of Sb-oxidizing bacteria (Jin et al., 2020), and the rate is promoted. Secondly, pH also affects the activities of antimony oxidase enzyme protein (Hamamura et al., 2013; Li, 2017). Thirdly, Sb(III) non-enzymatic reactions (H_2_O_2_-catalyzed reaction) for Sb-oxidizing bacteria is also a pH-dependent process. The half-life of 1 μmol/L H_2_O_2_ to oxidize Sb(III) is 117 and 11 days at pH of 8 and 9, respectively, while H_2_O_2_ can hardly oxidize Sb(III) at pH < 7.0; 50 μM H_2_O_2_ can completely oxidize 20 μM Sb(III) in 6 h at pH 9 (Leuz et al., 2006a; Kong et al., 2015). Fourthly, Sb(III) is more easily absorbed by various compounds, such as hydroxides of Fe, Mn, and Al; humic acid; and clay minerals in soil and water, and these compounds can induce catalytic oxidation of Sb(III) (Leuz et al., 2006b; Li, 2017), and the induction process is limited by pH. In summary, pH will affect the rate of Sb(III) oxidation by Sb-oxidizing bacteria from various aspects. This effect is a complex system and is still largely unknown.

##### Redox potential (Eh) or oxic–anoxic change

The optimal Eh for the growth of aerobic microorganisms is generally 300–400 mv, which also affects the transformation of Sb. Hockmann et al. (2014) reported that the transition to reducing conditions invoked by indigenous microbial activity at first led to the immobilization of Sb, as Sb(V) was reduced to Sb(III), which binded more extensively to iron (hydr)oxides, and the previously sorbed Sb(III) was gradually released into solution due to reductive dissolution of the iron(hydr)oxides if reducing conditions continued (Zhu et al., 2018; Burton et al., 2019). Markelova et al. (2018) also reported that the reductive precipitation of Sb(III) appears to be mainly microbially mediated during oxic–anoxic condition. It was previously clearly revealed that a majority of Sb(III) was kinetically oxidized into Sb(V) on surface of soils under aerobic conditions and a smaller amount of Sb(III) was oxidized into Sb(V) under anaerobic conditions (Cai et al., 2016). Several studies also have shown that the adsorbed Sb(III) by iron oxides [goethite, hydrated ferric oxide (HFO), and Fe(OH)_3_] or co-precipitated by FeCl_3_ was oxidized into Sb(V) under oxic conditions (Xi et al., 2013; Guo et al., 2014). It is widely known that iron/manganese oxide and microbes were omnipresent components in Sb-contaminated soils. However, to our knowledge, the oxidation behavior and mechanism of Sb(III) under coupling-mediated iron/manganese (hydr)oxides between microbes has not yet been described in literature. Therefore, the oxidation behavior of Sb-oxidizing bacteria induced by iron and manganese oxide is also a future research priority (Nguyen et al., 2017a, b; Li Y. et al., 2018).

##### Other factors

The oxidation rate of Sb(III) for various Sb-oxidizing bacteria has typically been determined in the laboratory using synthetic culture media, which is also held independent of other relevant factors. Therefore, studies of Sb(III) oxidation by Sb-oxidizing bacteria are relatively simple and controllable. However, the external soil environment contains a wide range of other components including high concentrations of nutrients such as nitrogen (N), phosphorus (P), potassium (K), and complex organic matter (including fulvic acid, humic acid, and low molecular organic matter) (Biver and Shotyk, 2012; Wu et al., 2019); trace elements; and a range of other biotic and abiotic phases and ions (Loni et al., 2020), such as iron/manganese (hydr)oxides (Levett et al., 2020), associated metal ions (Ca^2+^, Mg^2+^, As^3+^, and Cr^3+^) (Markelova et al., 2018), SO_4_^2+^/S^2–^ (Park et al., 2018; Zhu et al., 2018), and PO_4_^3+^ (Park et al., 2018), which affect the microbial oxidation of Sb(III). These chemical substances or ions may react with Sb(III) by complexation, adsorption, or induced catalysis, resulting in a greater degree of complexity in understanding and controlling the microbial oxidation process of Sb(III). There are still major challenges to understand the effects on the mechanism of the microbial oxidation of Sb(III).

### Mechanism for the Microbial Oxidation of Sb(III) by Sb-Oxidizing Bacteria

#### Enzyme Catalysis Reaction Mechanism

Because Sb(III) and As(III) may be biochemical analogs (Lehr et al., 2007), many researchers believe that As-oxidizing bacteria can also oxidize Sb(III) with the same oxidation mechanism in the early stage. With the deepening of research, people believe that the mechanisms of microbial oxidation of Sb(III) and As(III) are significantly different. Before 2007, *A. tumefaciens* sp. 5A that can oxidize As(III) and disruption of *aoxR* and *mrpB* mutants were all found to oxidize Sb(III) at the same rate by Lehr et al. (2007). Moreover, they also discovered that the expression of As(III) oxidase structural genes, *aoxAB*, was induced by As(III) but not by Sb(III). These findings first hinted that the Sb-oxidizing bacteria had independent Sb oxidation function genes and disparate Sb metabolism pathways. Subsequently, Wang et al. (2015) further found that disruption of *aioA*, which functions as an As(III) and Sb(III) oxidase in *A. tumefaciens* sp. 5A, caused only about 25% decrease of oxidation rate of Sb(III), and the expression of *aioBA* gene was induced by As(III) but not by Sb(III); thus, the author was convinced that more than one enzyme were involved in microbial oxidation of Sb(III). In 2013, six Sb-oxidizing bacteria without As(III) oxidation abilities were isolated from Xikuangshan by Li et al. (2013). Moreover, it was found that only a few Sb-oxidizing bacteria isolated from diverse mining areas contained As oxidase gene (Shi et al., 2013). The expression of *aioA* gene induced As(III) and Sb(III) in *Sinorhizobium*-related isolate strain, but it only possesses As(III) oxidation abilities without Sb(III) oxidation abilities (Hamamura et al., 2013). These findings indicate that microbial oxidation of As(III) and Sb(III) has different molecular mechanisms, and Sb-oxidizing bacteria contain antimony oxidation genes other than *aio*.

Luo et al. (2014) found that the abundances of genes (*aioA*, *arsC*, and *arrA*) and efflux protease (ArsB and ACR3) were positively correlated with the concentrations of As and Sb in soil with the help of real-time PCR technology. In 2015, some researchers (Xiong et al., 2011; Li et al., 2013) reported that the transcription expression of the iron–sulfur cluster gene *iscR* in *Comamonas* sp. S44 was reduced by Sb(III), and the activity of γ-glutamylcysteine ligase and the content of GSH were decreased, resulting in As(III), Cd(II), Cu(II), and H_2_O_2_ resistance and Sb(III) oxidation abilities declining in different degrees. Therefore, they were firmly convinced that *iscR* mainly *via* Fe–S cluster biogenesis and oxidative stress protection obtained Sb(III) resistance and oxidation ability. That same year, the As oxidation gene *aioA* was detected in *H. taeniospiralis* strain IDSBO-1 under anoxic conditions and *V. paradoxus* strain IDSBO-4 under aerobic conditions by Terry et al. (2015), but the expression of *aioA* was not induced by Sb(III). So, the authors speculated that the *aioA* gene was involved in Sb(III) oxidation *via* Sb(III)-specific pathway, and whether Arx and other oxidases were involved in Sb(III) oxidation remains to be confirmed. In 2017, As oxidation gene *aioA* was detected in *Shinella* sp. strain NLS1, which possesses As(III) and Sb(III) oxidation abilities under aerobic conditions (Nguyen et al., 2017b), but it was not detected in *Ensifer* sp. strain NLS4, which possesses Sb(III) oxidation abilities with nitrate as electron acceptor under anoxic conditions. These findings illustrated that the AioA enzyme played a vital role in Sb(III) and As(III) oxidation. It is important to note a new Sb(III) oxidase AnoA was discovered in *A. tumefaciens* GW4 and a comprehensive Sb(III) oxidation mechanism was proposed by Li (2017). The mechanism includes the components as follows: (1) the new Sb(III) oxidase AnoA catalyzes oxidation of Sb(III) with NADP^+^ as a coenzyme in bacterial cells. (2) The typical As oxidase AioAB catalyzes oxidation of Sb(III) in the periplasm. (3) Under Sb(III) stress, the oxidative stress in bacteria increased, resulting in generation of intracellular ROS and H_2_O_2_. (4) The absence of As oxidase AioAB causes up-regulated expression of Sb(III) oxidase AnoA antimony oxidase. (5) The absence of As oxidase AioAB causes increased content of H_2_O_2_ and up-regulated expression of *kata* gene in cells. (6) The H_2_O_2_ that induced under Sb(III) stress oxidizes Sb(III) to Sb(V). (7) The remaining H_2_O_2_ is degraded by catalase *KatA*. Therefore, the molecular mechanisms of the *A. tumefaciens* GW4 oxidation pathway for Sb(III) is a co-metabolic process, which includes intracellular enzymatic [Sb(III) oxidase AnoA and As oxidase AioAB] catalysis and oxidation by cellular H_2_O_2_ (Li et al., 2015, 2017). Moreover, the As oxidase AioA can affect the enzymatic reaction catalyzed by AnoA and the non-enzymatic reaction mediated by H_2_O_2_.

The *aioA* and *aioB* genes were detected in *Bosea* sp. AS-1 strains, which possess Sb(III) and As(III) oxidation abilities, but no *anoA* gene was detected (Lu et al., 2018). Meanwhile, *Bosea* sp. AS-1 exhibits diverse Sb(III) and As(III) oxidation abilities while having yeast or sodium acetate as a carbon source. Therefore, the authors speculated that there may be new oxidation genes except for *anoA* and *aioA* in *Bosea* sp. AS-1, and the specific gene types need to be further investigated. Subsequently, recent research (Gu et al., 2019, 2020) indicated that no AiOA and AnOA antimony oxidases were detected in *A. johnsonii* JH7, which possesses Sb(III) resistance and oxidation abilities, and the content of ArsH enzyme and ROS increased significantly under Sb(III) stress. Therefore, it is speculated that the mechanism of *A. johnsonii* JH7 oxidation of Sb(III) may be associated to ArsH enzyme and ROS. In 2020, a new research (Sun et al., 2020) found that the oxidation rate of Sb(III) was faster than that of As for *R. rhizosphaerae* strain YW11, and Sb(III) and As(III) can induce the expression of *aioAB* As oxidation gene. However, *R. rhizosphaerae* strain YW11 lost Sb(III) or As(III) oxidation ability after disruption of *aioA* gene, but its Sb(III) resistance still existed. These latest findings support that intracellular enzymatic (AioA) catalysis oxidation of Sb(III) is the only molecular mechanism for *R. rhizosphaerae* strain YW11, but there are multiple Sb(III)-resistant mechanisms.

#### The Non-enzymatic Catalysis Reaction Mechanism

In addition to genes directly related to the Sb(III) and As(III) oxidation, some genes have non-specific oxidation effects on low-valence elements such as Sb(III) and As(III), such as enzymes and genes related to H_2_O_2_. They regulate the production, accumulation, consumption, and efflux of H_2_O_2_ in cells and immediately affect the Sb(III) oxidation. This abiotic oxidation of H_2_O_2_ may play a dominant role in some Sb-oxidizing bacteria oxidation mechanism.

The toxicity of Sb(III) affects the electron transport on the bacterial respiratory chain and disrupts the redox balance in the cell (Ma et al., 1999), thereby inducing the bacteria to produce ROS, the types incorporate superoxide anion (O_2_−), hydroxyl radical (OH), hydroperoxy radical (HO_2_), hydrogen peroxide (H_2_O_2_), and various peroxides (Lushchak, 2014). Some bacteria have evolved a series of defense mechanisms, including specific enzymes and non-enzymatic antioxidants, to resist ROS toxicity and oxidative stress in cells. The specific enzymes primarily include superoxide dismutase (Sod) [the types of Sod are as follows: Fe type, Mn type, Cu–Zn type, and Sod with Ni as a cofactor (Fridovich, 1995); catalase/peroxidase (Lushchak, 2014), peroxide reductase (Prxs), or alkyl hydrogen peroxide reductase (Ahps); glutathione reduced protein; and glutathione reductase]. Non-enzymatic antioxidants include reduced coenzyme II, ascorbic acid, β-carotene, α-tocopherol, and GSH (Calderón et al., 2009). ROS produces H_2_O_2_ and O_2_ under the catalysis of Sod, and H_2_O_2_ will result in Fenton reaction with free iron ions in cells to cause damage to DNA, proteins, and lipids and generate cell death (Touati, 2000; Wei et al., 2019). Meanwhile, H_2_O_2_ can directly oxidize Sb(III) and reduce its toxicity (Quentel et al., 2006). Therefore, as a non-enzymatic reaction, H_2_O_2_ can simultaneously reduce the dual toxicity caused by Sb(III) and cell oxidative stress. It plays a crucial role in regulating the Sb resistance and Sb-oxidizing bacteria oxidation.

**FIGURE 6:**
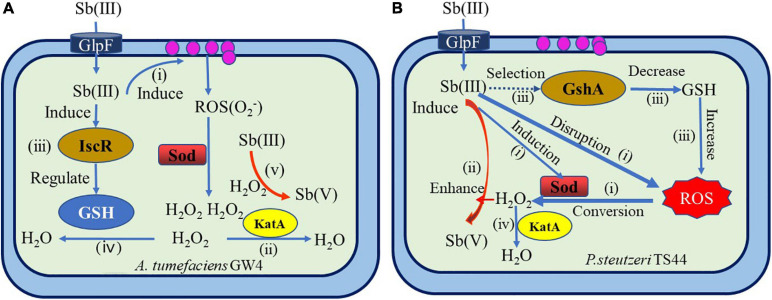
Overview of mechanisms of bacterial antimonite oxidation (Liu et al., 2015; Wang et al., 2016; Li, 2017). **(A)** A hypothetical model of IscR’s regulation of bacterial Sb(III) oxidation in *A. tumefaciens* GW4. (i) Sb(III) induced the production of H_2_O_2_ *via* the bacterial oxidative stress response. (ii) H_2_O_2_ was partially consumed by catalase *KatA*. (iii) Sb(III) induced the expression of (Fe–S) assembly transcription factor IscR, which could positively contribute to GSH formation. (iv) Then, H_2_O_2_ was partially consumed by GSH. (v) Subsequently, H_2_O_2_ oxidized Sb(III) to Sb(V). **(B)** The proposed model for Sb(III) bacterial oxidation in *P. stutzeri* TS44. (i) the addition of Sb(III) would trigger the ROS-protective system by inducing the transcription of *sodB*, *sodC*, and *katE*, with *SodB* and *SodC* catalyzing the conversion of ROS to H_2_O_2_, while *KatE* is responsible for the degradation of excessive H_2_O_2_; (ii) the increased cellular H_2_O_2_ content enhanced the Sb(III) oxidation rate; (iii) the addition of Sb(III) played a selection role on the characterization of *gshA* insertion; (iv) the accumulated H_2_O_2_ is partially consumed by the upregulated catalase *KatE*.

In recent years, systematic studies on the non-enzymatic antimony oxidation reaction process and mechanism of Sb-oxidizing bacteria, such as *A. tumefaciens* GW4 and *P. stutzeri* TS44 (Liu et al., 2015; Wang et al., 2016; Li, 2017; Figure 6), include the following aspects: The increase of Sb(III) in the environment will trigger the ROS protection system of bacteria and then induce the transcriptional expression of the *sodB*, *sodC*, and *katE* genes related to oxidative stress. The function of *sodB* and *sodC* is to catalyze the conversion of ROS into H_2_O_2_, and the function of *KatE* is to consume excessive H_2_O_2_. The increase of H_2_O_2_ content in the cell promotes the oxidation rate of Sb(III). Sb(III) can induce the expression of gshA enzyme or (Fe–S) assembly IscR transcription factor; IscR or gshA controls the synthesis of GHS. The excess H_2_O_2_ in the cell is consumed by *KatE*, which is induced by Sb(III), thereby protecting the cell. Therefore, bacteria that depend on the ROS protection mechanism produced by GHS can promote the oxidation rate of Sb(III), and GHS, ROS, and H_2_O_2_ play a crucial role in the non-enzymatic oxidation of Sb(III) by microbes. However, due to the complex types of ROS in the environment and the diversity of Sod enzymes, the regulatory relationship between GHS, ROS, and H_2_O_2_ in various bacteria under Sb(III) stress is not completely clear, and further research is needed.

#### The Molecular Mechanism of Sb-Oxidizing Bacteria Oxidizing Sb(III)

**FIGURE 7:**
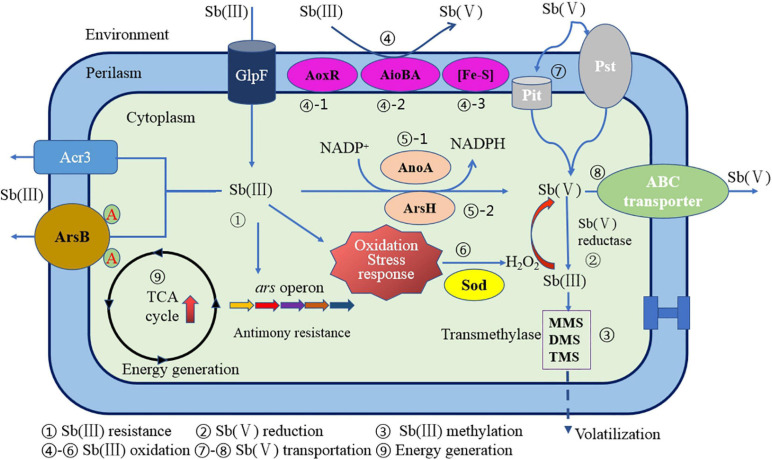
A schematic diagram of Sb resistance of Sb-oxidizing bacteria and Sb(III) oxidation mechanism (Li, 2017).

In summary, the oxidation mechanism for a particular Sb(III) oxidase and gene regulation has become more and more unambiguous. However, the microbial oxidation mechanism of Sb(III) is the result of the combined effect of enzymatic oxidation mechanism and non-enzymatic oxidation mechanism. It is still arduous to systematically and comprehensively analyze the Sb oxidation mechanism under these circumstances. A relatively complete Sb(III) oxidation mechanism and mode for Sb-oxidizing bacteria have been proposed by some researchers (Li, 2017). As shown in Figure 7, the Sb(III) oxidation mechanism mainly includes the following: (1) Sb(III) resistance; (2) Sb(V) reduction; (3) Sb(III) methylation; (4) Sb(III) oxidation by AoxR, AioBA, and (Fe–S) oxidase; (5) Sb(III) oxidation by AnoA or ArsH enzyme; (6) H_2_O_2_ production and non-enzymatic Sb(III) oxidation; (7) Sb(V) uptake; (8) Sb(V) transportation; and (9) energy generation and other biochemical reaction processes. So, the Sb oxidation gene–enzyme system specific in Sb-oxidizing bacteria cell principally includes *aox*R–AoxR, *aioA*–AioA, *anoA*–AnoA, *iscR*–IscR, and *arsH*–ArsH. Except these Sb(III) oxidases, other genes or enzymes required for Sb(III) oxidation are still unknown. The non-specific oxidation system is principally composed of GHS, ROS, and H_2_O_2_, which constitute the oxidative pressure regulation mechanism for Sb-oxidizing bacteria. It can be seen that the Sb(III) oxidation for Sb-oxidizing bacteria is controlled by non-enzymatic and enzymatic reactions mediated by H_2_O_2_. Due to the diversity of Sb oxidase, there may be various Sb(III) oxidases involved in Sb(III) oxidation reaction. However, compared to As oxidase system, our understanding of types of Sb(III) oxidases and its molecular mechanism remains incomplete. Therefore, it is urgent to carry out in-depth research from the perspectives of metagenomics, transcriptomics, proteomics, and enzyme genomics in order to fully reveal the molecular mechanism of microbial oxidation of Sb(III).

## Potential Application of Sb-Oxidizing Bacteria in the Remediation and Treatment of Sb-Contaminated Environment

There are many mechanisms of bioremediation for potentially toxic element pollution. Generally, *prokaryotes* can reduce uptake and increase efflux heavy metal to achieve concentration control. As well, *eukaryotes* can detoxify by chelating their metabolites with heavy metal ions. Many bacteria evolved with diverse metabolic abilities, including oxidation–reduction and the second mineralization, reducing a variety of toxic metals and showing an important potential application in the bioremediation of contaminated environment (Gaoshang et al., 2012; Yao et al., 2020). To our knowledge, typical Sb-oxidizing bacteria, such as *Pseudomonas*, *Comamonas*, *Acinetobacter*, and *Sphingomonadaceae*, are widely distributed in the Sb-contaminated soil environment, but application of Sb-oxidizing bacteria in Sb pollution remediation has not yet been described in literature (Doherty et al., 2017; Nakamaru and Martín Peinado, 2017). Remediation of the Sb-polluted soil by the Sb-oxidizing bacteria has many advantages, such as simple living conditions, good Sb(III) oxidation performance and detoxification, and environmental friendliness (Na, 2007; Yu, 2016). So, it can be expected that in the near future, Sb-oxidizing bacteria will play a crucial role in the remediation of Sb pollution. In addition, many Sb-oxidizing bacteria also exhibit excellent adsorption (Jin et al., 2020), complexation, and secondary mineralization (Loni et al., 2020) during the remediation process. So, the application of Sb-oxidizing bacteria in bioremediation technology is not a single oxidation and detoxification mechanism (Verbeeck et al., 2020) but a combination of biological, chemical, and physical mechanisms (De-Ju et al., 2016; Yu, 2016; Urík et al., 2019). Simultaneously, the development of potentially toxic element pollution bioremediation technology tends to be combined remediation technology (Liu et al., 2019; Qiu et al., 2019). Usually, the process flow combines multiple remediation technologies to deliver preferred remediation effects (Jumei et al., 2019; Lu et al., 2019), such as microbial-phytoremediation technology (Nie et al., 2017; Jumei et al., 2019; Long et al., 2019; Lu et al., 2019; Ma et al., 2019; Sun et al., 2019; Zhang and Hu, 2019), which can take into account the advantages of both, has become a hotspot of bioremediation technology internationally. All in all, if the mechanism of antimony oxidation by Sb-oxidizing bacteria and various optimal factors is explored and perfected, it provides an excellent prospect for wide-scale application.

## Conclusion and Prospects

Antimony and its compounds are potentially toxic carcinogens that have the characteristics of high toxicity, great damage, and long-distance migration, so its geomicrobiological behavior has considerable attention from the research community and government stakeholders. As a strategic resource and industrial raw material, Sb supply from mining is still significant, resulting in millions of tons of Sb-bearing waste rocks produced annually. An important aspect of its geomicrobiological behavior is the dissolution and oxidation of Sb(III) from Sb-bearing waste rocks. The majority of previous research has focused on the abiotic dissolution and oxidation process of Sb(III), neglecting the mediating role of microbes. On the one hand, microbiota interact with the dissolution, oxidation, reduction, methylation, bioaccumulation, and mobility of Sb, affecting the ultimate fate of Sb in the environment. While on the other, Sb also affects the microbial activity of the biomass, respiration rate, enzyme activity, community structure, and biochemical processes and generates ecological effects. The oxidation process mediated by microbes plays an important role in Sb speciation, mobility, and bioavailability in the natural environment and potentially provides an environmentally friendly and efficient Sb pollution remediation method. So far, more than 90 strains of antimony-oxidizing bacteria had been identified, of which 97.7% belong to the phylum *Proteobacteria*, mainly distributed in *Pseudomonas*, *Comamonas*, and *Acinetobacter.* The microbial metabolism model for Sb has been initially proposed, including a variety of biochemical process, such as Sb(III) resistance, Sb(V) reduction, Sb(III) methylation, enzymatic Sb(III) oxidation, non-enzymatic Sb(III) oxidation, Sb(V) uptake, Sb(V) transportation and energy generation, and Sb(III) oxidase including AoxR, AioA, AnoA, IscR, and ArsH. The non-enzymatic Sb(III) oxidation is regulated by GHS, ROS, and H_2_O_2_, which dominate the oxidative pressure regulation mechanism. Due to the diversities and complexity of the genes and enzymes, microbial Sb(III) oxidation mechanisms are still unclear and need to be studied further.

In order to understand of role that microbes play in the biogeochemistry cycling of and provide a potential approach for environmental Sb bioremediation, we recommend that the following aspects should be further studied: (1) more Sb-oxidizing bacteria, especially autotrophic Sb-oxidizing bacteria, should be isolated and screened from the environment to establish a rich and diverse resource of Sb-oxidizing bacteria. (2) The genes or enzymes required for Sb(III) oxidation are still unknown. It is urgent to investigate from the perspectives of metagenomics, transcriptomics, proteomics, and enzyme genomics to fully illustrate the microbial Sb(III) oxidation mechanisms. (3) Based on proteomics and enzyme genomics, the regulatory function of non-enzymatic reaction genes (such as *katA*) in Sb-oxidizing bacteria should be studied in depth to understand the interaction between enzymatic and non-enzymatic Sb(III) oxidation mechanisms and reveal the relationship and difference between the Sb(III) oxidation and Sb(III) resistance mechanism of Sb-oxidizing bacteria. (4) To our knowledge, the pathway for Sb(V) uptake and Sb(V) efflux for Sb-oxidizing bacteria is still unknown. Since the phosphate-binding protein PstS2 is induced by Sb(V) (Gu et al., 2019, 2020), it is recommended to combine the phosphate transport system of Sb-oxidizing bacteria to elucidate the transport mechanism of Sb(V) uptake and Sb(V) efflux. (5) The Sb-oxidizing bacteria with strong Sb(III) resistance and oxidation ability should be selected using genetic engineering technology, and bioremediation technology and the mechanism of microbial and higher plants also should be studied.

## Author Contributions

RD conceived and designed the study. YC, XD, SZ, BR, and GJ analyzed the data. RD, YC, and ZH wrote the manuscript. AH assessed the data and reviewed the manuscript. All authors read and approved the manuscript.

## Conflict of Interest

GJ is employed by Hsikwangshan Twinkling Star Co., Ltd. The remaining authors declare that the research was conducted in the absence of any commercial or financial relationships that could be construed as a potential conflict of interest.

## Publisher’s Note

All claims expressed in this article are solely those of the authors and do not necessarily represent those of their affiliated organizations, or those of the publisher, the editors and the reviewers. Any product that may be evaluated in this article, or claim that may be made by its manufacturer, is not guaranteed or endorsed by the publisher.
