# Supplementary material for: A Critical Review of Resistance and Oxidation Mechanisms of Sb-Oxidizing Bacteria for the Bioremediation of Sb(III) Pollution
Source: Front Microbiol. 2021 Sep 7;12:738596. doi: 10.3389/fmicb.2021.738596 (PMC8453088; doi:10.3389/fmicb.2021.738596)
Supplement: Supplementary file 1 [file Table_1.doc]

Tab.S 1 Media and composition for screening antimony oxidising bacteria

Commonly used media can be divided into carbon source-containing media and carbon source-free media, and the composition is as follows.

(1) The composition of CDM: MgSO_4_·7H_2_O 2 g, NH_4_Cl 1g, Na_2_SO_4_ 1 g, K_2_HPO_4_ 0.013 g, Ca_2_Cl_2_·2H_2_O 0.067 g, Na-lactate 5 g, agar 15 g, distilled water 1000 mL, pH=7.2.(Shi et al.,2013)

(2) The composition of M-CDM: KH_2_PO_4_ 0.1 g, NH_4_Cl 0.1 g, CaCl_2_ 0.01 g, KCl 0.05 g, NaHCO_3_ 0.5 g, yeast extract 1 g, and 1 mL/L of vitamin solution, distilled water 1000 mL. The concentration of Sb(Ⅲ) in the enrichment medium was fixed at 500 mmol/L by adding potassium antimony(III) tartrate (KSbC_4_H_4_O_7_·0.5H_2_O).(Nguyen et al.,2017)

(3) The composition of CDM-A: K_2_HPO_4_ 0.225 g, KH_2_PO_4_ 0.225 g, NaCl 0.46 g, (NH_4_)_2_SO_4_ 0.225 g, MgSO_4_·7H_2_O 0.117 g, yeast extract 1.0 g, Na lactate 2.24 g , NaNO_3_ 1.7 g, NaHCO_3_ 4.2 g, Na_2_S·9H_2_O 0.1 g, and cysteine-HCl (0.1) (all in gramsper liter), SL10 trace element solution (1.0), distilled water 1000 mL.(Terry et al.,2015)

(4) The composition of Beef extract peptone AGAR medium: peptone 10 g, beef extract 3 g, NaCl 5 g, pH=7.0.(Du et al.,2020)

(5) The composition of LCM/HCM enrichment Medium: 120 mM HCO_3_^−^ and 0.002% (wt/vol) yeast extract, distilled water 1000 mL; the medium was amended with 100 μM Sb(Ⅲ) (as potassium antimonyl tartrate).(Hamamura et al.,2013)

(6) The composition of 9K Medium: (NH_4_)_2_SO_4_ 3.0 g, KH_2_PO_4_ 0.5 g, KCl 0.1 g, MgSO_4_·7H_2_O 0.5 g, Ca(NO_3_)_2_·2H_2_O 0.01 g, FeSO_4_·7H_2_O 44.2 g, distilled water 1000 mL.(Tsaplina et al.,2010)

**References:**

Du H., Liu X., Tao J., Lei M., Liu Y., Yang R,,Tie B..(2020). Screening of three antimony-resistant soil bacteria and their adsorption property for antimony. *Acta Scientiae Circumstantiae*，40( 6) : 2205-2211 (In Chinese). doi: 10.13671/j.hjkxxb.2020.0087

Hamamura, N., Fukushima, K., Itai, T. (2013). Identification of antimony- and arsenic-oxidizing bacteria associated with antimony mine tailing. *Microbes Environ.* 28(2), 257-263. doi: 10.1264/jsme2.ME12217

Nguyen, V. K., Choi, W., Yu, J., Lee, T. (2017). Microbial oxidation of antimonite and arsenite by bacteria isolated from antimony-contaminated soils. *Int. J. Hydrogen Energ.* 42(45), 27832-27842. doi: 10.1016/j.ijhydene.2017.08.056

Shi, Z., Cao, Z., Qin, D., Zhu, W., Wang, Q., and Li, M., et al. (2013). Correlation models between environmental factors and bacterial resistance to antimony and copper. *PLoS One*. 8(10), e78533. doi: 10.1371/journal.pone.0078533

Terry, L. R., Kulp, T. R., Wiatrowski, H., Miller, L. G., Oremland, R. S. (2015). Microbiological oxidation of antimony(iii) with oxygen or nitrate by bacteria isolated from contaminated mine sediments. *Appl. Environ. Microb.* 81(24), 8478-8488. doi: 10.1128/AEM.01970-15

Tsaplina, I. A., Zhuravleva, A. E., Belyi, A. V., Kondrat Eva, T. F. (2010). Functional diversity of an aboriginal microbial community oxidizing the ore with high antimony content at 46–47°C. *Microbiology*. 79(6), 735-746. doi: 10.1134/S0026261710060032
