# Supplementary material for: A Critical Review of Resistance and Oxidation Mechanisms of Sb-Oxidizing Bacteria for the Bioremediation of Sb(III) Pollution
Source: Front Microbiol. 2021 Sep 7;12:738596. doi: 10.3389/fmicb.2021.738596 (PMC8453088; doi:10.3389/fmicb.2021.738596)
Supplement: Supplementary file 2 [file Table_2.doc]

Tab.S2 The reported bioinformatics taxonomy of antimony oxidising bacteria

| NO. | Name（Genbank NO.） | Phyla | Classes | Orders | Families | Genera | As oxidizing ability |
| --- | --- | --- | --- | --- | --- | --- | --- |
| 1 | *Sphingopyxis* sp. DA6 (KC294091) | *Proteobacteria* | *Alphaproteobacteria* | *Sphingomonadales* | *Sphingomonadaceae* | *Sphingopyxis* | - |
| 2 | *Paracoccus* sp. JC6 (KC294044) | *Proteobacteria* | *Alphaproteobacteria* | *Rhodobacterales* | *Rhodobacteraceae* | *Paracoccus* | - |
| 3 | *Aminobacter* sp. LS5 (KC294021) | *Proteobacteria* | *Alphaproteobacteria* | *Rhizobiales* | *Phyllobacteriaceae* | *Aminobacter* | - |
| 4 | *Rhizobium* sp. NT-26 (AF159453) | *Proteobacteria* | *Alphaproteobacteria* | *Rhizobiales* | *Rhizobiaceae* | *Rhizobium* | Yes |
| 5 | *Agrobacterium tumefaciens* strain 42 (AF388030) | *Proteobacteria* | *Alphaproteobacteria* | *Rhizobiales* | *Rhizobiaceae* | *Rhizobium* | Yes |
| 6 | *Shinella* sp. strain NLS1 (KY594721) | *Proteobacteria* | *Alphaproteobacteria* | *Rhizobiales* | *Rhizobiaceae* | *Shinella* | - |
| 7 | *Ensifer* sp. strain NLS4 (KY594722) | *Proteobacteria* | *Alphaproteobacteria* | *Rhizobiales* | *Rhizobiaceae* | *Ensifer* | - |
| 8 | *Ensifer adhaerens* strain WJB36 (KU877637) | *Proteobacteria* | *Alphaproteobacteria* | *Rhizobiales* | *Rhizobiaceae* | *Ensifer* | - |
| 9 | *Ensifer* sp. WXBRA3 (KJ184916) | *Proteobacteria* | *Alphaproteobacteria* | *Rhizobiales* | *Rhizobiaceae* | *Ensifer* | - |
| 10 | *Sinorhizobium* sp. B84 (HQ877776) | *Proteobacteria* | *Alphaproteobacteria* | *Rhizobiales* | *Rhizobiaceae* | *Sinorhizobium* | - |
| 11 | *Sinorhizobium* sp. IK-A2 (KC012938) | *Proteobacteria* | *Alphaproteobacteria* | *Rhizobiales* | *Rhizobiaceae* | *Sinorhizobium* | Yes |
| 12 | *Shinella fusca* strain DC-196 (NR_116889) | *Proteobacteria* | *Alphaproteobacteria* | *Rhizobiales* | *Rhizobiaceae* | *Shinella* | - |
| 13 | *Shinella* sp. C72 (KT361091) | *Proteobacteria* | *Alphaproteobacteria* | *Rhizobiales* | *Rhizobiaceae* | *Shinella* | - |
| 14 | *Comamonas* sp. S44 (FJ210285) | *Proteobacteria* | *Betaproteobacteria* | *Burkholderiales* | *Comamonadaceae* | *Comamonas* | NO |
| 15 | *Hydrogenophaga* sp. IDSBO-1 (KM199760) | *Proteobacteria* | *Betaproteobacteria* | *Burkholderiales* | *Comamonadaceae* | *Hydrogenophaga* | Yes |
| 16 | *Variovorax* sp. IDSBO-4 (KM199761) | *Proteobacteria* | *Betaproteobacteria* | *Burkholderiales* | *Comamonadaceae* | *Variovorax* | Yes |
| 17 | *Thiobacillus denitrificans* strain NCIMB 9548 (NR025358) | *Proteobacteria* | *Betaproteobacteria* | *Nitrosomonadales* | *Thiobacillaceae* | *Thiobacillus* | - |
| 18 | *Comamonas* sp. JL25 (JF740042) | *Proteobacteria* | *Betaproteobacteria* | *Burkholderiales* | *Comamonadaceae* | *Comamonas* | - |
| 19 | *Comamonas* sp. JL40 (JF740043) | *Proteobacteria* | *Betaproteobacteria* | *Burkholderiales* | *Comamonadaceae* | *Comamonas* |  |
| 20 | *Variovora*x sp. JL23 (JF740055) | *Proteobacteria* | *Betaproteobacteria* | *Burkholderiales* | *Comamonadaceae* | *Variovorax* | - |
| 21 | *Comamonas* sp. NL11 (KF264577) | *Proteobacteria* | *Betaproteobacteria* | *Burkholderiales* | *Comamonadaceae* | *Comamonas* | - |
| 22 | *Cupriavidus* sp. NL4 (KF264570) | *Proteobacteria* | *Betaproteobacteria* | *Burkholderiales* | *Burkholderiaceae* | *Cupriavidus* | - |
| 23 | *Proteus cibarius strain* DSHN0704 (MH613348) | *Proteobacteria* | *Gammaproteobacteria* | *Enterobacterales* | *Morganellaceae* | *Proteus* | - |
| 24 | *Pseudomonas* sp. NL2 (KF264568) | *Proteobacteria* | *Gammaproteobacteria* | *Pseudomonadales* | *Pseudomonadaceae* | *Pseudomonas* | - |
| 25 | *Acinetobacter* sp. NL1 (KF264567) | *Proteobacteria* | *Gammaproteobacteria* | *Pseudomonadales* | *Moraxellaceae* | *Acinetobacter* | - |
| 26 | *Stenotrophomonas maltophilia* 6B2-1 (AY445079) | *Proteobacteria* | *Gammaproteobacteria* | *Xanthomonadales* | *Xanthomonadaceae* | *Stenotrophomonas* | - |
| 27 | *Acinetobacter* sp. JL7 (JF740033) | *Proteobacteria* | *Gammaproteobacteria* | *Pseudomonadales* | *Moraxellaceae* | *Acinetobacter* | - |
| 28 | *Stenotrophomonas* sp. IK-S2 (KC012941) | *Proteobacteria* | *Gammaproteobacteria* | *Xanthomonadales* | *Xanthomonadaceae* | *Stenotrophomonas* | - |
| 29 | *Acinetobacter* sp. NL12 (KF264578) | *Proteobacteria* | *Gammaproteobacteria* | *Pseudomonadales* | *Moraxellaceae* | *Acinetobacter* | - |
| 30 | *Pseudomona*s sp. NL5 (KF264571) | *Proteobacteria* | *Gammaproteobacteria* | *Pseudomonadales* | *Pseudomonadaceae* | *Pseudomonas* | - |
| 31 | *Pseudomonas* sp. NL6 (KF264572) | *Proteobacteria* | *Gammaproteobacteria* | *Pseudomonadales* | *Pseudomonadaceae* | *Pseudomonas* | - |
| 32 | *Pseudomonas* sp. NL10 (KF264576) | *Proteobacteria* | *Gammaproteobacteria* | *Pseudomonadales* | *Pseudomonadaceae* | *Pseudomonas* | - |
| 33 | *Pseudomonas* sp. IK-S1 (KC012939) | *Proteobacteria* | *Gammaproteobacteria* | *Pseudomonadales* | *Pseudomonadaceae* | *Pseudomonas* | - |
| 34 | *Stenotrophomonas* sp. JL9 (JF740054) | *Proteobacteria* | *Gammaproteobacteria* | *Xanthomonadales* | *Xanthomonadaceae* | *Stenotrophomonas* | - |
| 35 | *Janibacter* sp. LH2 (KC294028) | *Actinobacteria* | *Actinobacteria* | *Micrococcales* | *Intrasporangiaceae* | *Janibacter* | - |
| 36 | *Acidithiobacillus ferridurans* strain ATCC 33020 (AF173880) | *Proteobacteria* | *Acidithiobacillia* | *Acidithiobacillales* | *Acidithiobacillaceae* | *Acidithiobacillus* | - |
| 37 | *Flavihumibacter stibioxidans* strain YS-17 (KX014806) | *Bacteroidetes* | *Chitinophagia* | *Chitinophagales* | *Chitinophagaceae* | *Flavihumibacter* | - |
| 38 | *Bosea* sp. AS-1 (CP022372) | *Proteobacteria* | *Alphaproteobacteria* | *Rhizobiales* | *Bradyrhizobiaceae* | *Bosea* | Yes |
| 39 | *Acinetobacter* sp. JH7(MK294307) | *Proteobacteria* | *Gammaproteobacteria* | *Pseudomonadales* | *Moraxellaceae* | *Acinetobacter* | - |
| 40 | *Agrobacterium tumefaciens* GW4 (DQ647054) | *Proteobacteria* | *Alphaproteobacteria* | *Rhizobiales* | *Rhizobiaceae* | *Rhizobium* | - |
| 41 | *Agrobacterium tumefaciens* A5(MF156930) | *Proteobacteria* | *Alphaproteobacteria* | *Rhizobiales* | *Rhizobiaceae* | *Rhizobium* | Yes |
| 42 | *Sinorhizobium* sp. GW(DQ337550) | *Proteobacteria* | *Alphaproteobacteria* | *Rhizobiales* | *Rhizobiaceae* | *Sinorhizobium* | - |
| 43 | *Paraccocus versutus* XT0.6 | *Proteobacteria* | *Alphaproteobacteria* | *Rhodobacterales* | *Rhodobacteraceae* | *Paracoccus* | - |
| 44 | *Agrobacterium tumefaciens* (ASM97156v1) | *Proteobacteria* | *Alphaproteobacteria* | *Rhizobiales* | *Rhizobiaceae* | *Rhizobium* | - |
| 45 | *Peseudomanas stutzeri* TS44(EU073110) | *Proteobacteria* | *Gammaproteobacteria* | *Pseudomonadales* | *Pseudomonadaceae* | *Pseudomonas* | - |
| 46 | *Ichinokawa isolates* strain 33 (NR041577) | *Proteobacteria* | *Gammaproteobacteria* | *Xanthomonadales* | *Xanthomonadaceae* | *Stenotrophomonas* | - |
| 47 | *Variovorax* sp. LS1（KC294017） | *Proteobacteria* | *Betaproteobacteria* | *Burkholderiales* | *Comamonadaceae* | *Variovorax* | - |
| 48 | *Comamonas* sp. DF1（KC294052） | *Proteobacteria* | *Betaproteobacteria* | *Burkholderiales* | *Comamonadaceae* | *Comamonas* | - |
| 49 | *Comamonas* sp. DF2（KC294053） | *Proteobacteria* | *Betaproteobacteria* | *Burkholderiales* | *Comamonadaceae* | *Comamonas* | - |
| 50 | *Comamonas* sp. DS1（KC294078） | *Proteobacteria* | *Betaproteobacteria* | *Burkholderiales* | *Comamonadaceae* | *Comamonas* | - |
| 51 | *Comamonas* sp. JC9（KC294047） | *Proteobacteria* | *Betaproteobacteria* | *Burkholderiales* | *Comamonadaceae* | *Comamonas* | - |
| 52 | *Comamonas* sp. JC12（KC294050） | *Proteobacteria* | *Betaproteobacteria* | *Burkholderiales* | *Comamonadaceae* | *Comamonas* | - |
| 53 | *Comamonas* sp. JC13（KC294051） | *Proteobacteria* | *Betaproteobacteria* | *Burkholderiales* | *Comamonadaceae* | *Comamonas* | - |
| 54 | *Acinetobacter* sp. DS2（KC294079） | *Proteobacteria* | *Gammaproteobacteria* | *Pseudomonadales* | *Moraxellaceae* | *Acinetobacter* | - |
| 55 | *Acinetobacter* sp. LH3（KC294029） | *Proteobacteria* | *Gammaproteobacteria* | *Pseudomonadales* | *Moraxellaceae* | *Acinetobacter* | - |
| 56 | *Acinetobacter* sp. LH4（KC294030） | *Proteobacteria* | *Gammaproteobacteria* | *Pseudomonadales* | *Moraxellaceae* | *Acinetobacter* | - |
| 57 | *Acinetobacter* sp. DC2（KC294067） | *Proteobacteria* | *Gammaproteobacteria* | *Pseudomonadales* | *Moraxellaceae* | *Acinetobacter* | - |
| 58 | *Pseudomonas* sp. DS7（KC294084） | *Proteobacteria* | *Gammaproteobacteria* | *Pseudomonadales* | *Pseudomonadaceae* | *Pseudomonas* | - |
| 59 | *Pseudomonas* sp. DF5（KC294056） | *Proteobacteria* | *Gammaproteobacteria* | *Pseudomonadales* | *Pseudomonadaceae* | *Pseudomonas* | - |
| 60 | *Pseudomonas* sp. DF7（KC294058） | *Proteobacteria* | *Gammaproteobacteria* | *Pseudomonadales* | *Pseudomonadaceae* | *Pseudomonas* | - |
| 61 | *Pseudomonas* sp. DS4（KC294081） | *Proteobacteria* | *Gammaproteobacteria* | *Pseudomonadales* | *Pseudomonadaceae* | *Pseudomonas* | - |
| 62 | *Pseudomonas* sp. DA5（KC294090） | *Proteobacteria* | *Gammaproteobacteria* | *Pseudomonadales* | *Pseudomonadaceae* | *Pseudomonas* | - |
| 63 | *Pseudomonas* sp. DF12（KC294063） | *Proteobacteria* | *Gammaproteobacteria* | *Pseudomonadales* | *Pseudomonadaceae* | *Pseudomonas* | - |
| 64 | *Pseudomonas* sp. DA2（KC294087） | *Proteobacteria* | *Gammaproteobacteria* | *Pseudomonadales* | *Pseudomonadaceae* | *Pseudomonas* | - |
| 65 | *Pseudomonas* sp. DF11（KC294062） | *Proteobacteria* | *Gammaproteobacteria* | *Pseudomonadales* | *Pseudomonadaceae* | *Pseudomonas* | - |
| 66 | *Pseudomonas* sp. DF3（KC294054） | *Proteobacteria* | *Gammaproteobacteria* | *Pseudomonadales* | *Pseudomonadaceae* | *Pseudomonas* | Yes |
| 67 | *Pseudomonas* sp. DF9（KC294060） | *Proteobacteria* | *Gammaproteobacteria* | *Pseudomonadales* | *Pseudomonadaceae* | *Pseudomonas* |  |
| 68 | *Pseudomonas* sp. DC5（KC294070） | *Proteobacteria* | *Gammaproteobacteria* | *Pseudomonadales* | *Pseudomonadaceae* | *Pseudomonas* | - |
| 69 | *Pseudomonas* sp. DC8（KC294073） | *Proteobacteria* | *Gammaproteobacteria* | *Pseudomonadales* | *Pseudomonadaceae* | *Pseudomonas* | - |
| 70 | *Pseudomonas* sp. JC11（KC294049） | *Proteobacteria* | *Gammaproteobacteria* | *Pseudomonadales* | *Pseudomonadaceae* | *Pseudomonas* | - |
| 71 | *Pseudomonas* sp. TC13（KC294138） | *Proteobacteria* | *Gammaproteobacteria* | *Pseudomonadales* | *Pseudomonadaceae* | *Pseudomonas* | - |
| 72 | *Pseudomonas* sp. DC7（KC294072） | *Proteobacteria* | *Gammaproteobacteria* | *Pseudomonadales* | *Pseudomonadaceae* | *Pseudomonas* | - |
| 73 | *Pseudomonas* sp. DF8（KC294059） | *Proteobacteria* | *Gammaproteobacteria* | *Pseudomonadales* | *Pseudomonadaceae* | *Pseudomonas* | - |
| 74 | *Pseudomonas* sp. DA4（KC294089） | *Proteobacteria* | *Gammaproteobacteria* | *Pseudomonadales* | *Pseudomonadaceae* | *Pseudomonas* | - |
| 75 | *Paracoccus* sp. LH8（KC294034） | *Proteobacteria* | *Alphaproteobacteria* | *Rhodobacterales* | *Rhodobacteraceae* | *Paracoccus* | - |
| 76 | *Sphingopyxis* sp. DS8（KC294085） | *Proteobacteria* | *Alphaproteobacteria* | *Sphingomonadales* | *Sphingomonadaceae* | *Sphingopyxis* | NO |
| 77 | *Bacillus* sp. DF4（KC294055） | *Firmicutes* | *Bacilli* | *Bacillales* | *Bacillaceae* | *Bacillus* | - |
| 78 | *Arthrobacter* sp. LH11（KC294037） | *Actinobacteria* | *Actinobacteria* | *Micrococcales* | *Micrococcaceae* | *Arthrobacter* | - |
| 79 | *Pseudomonas* sp.AO-1 (MN720563) | *Proteobacteria* | *Gammaproteobacteria* | *Pseudomonadales* | *Pseudomonadaceae* | *Pseudomonas* | - |
| 80 | *Pseudarthrobacter* sp. AO-2 (MN720564) | *Actinobacteria* | *Actinobacteria* | *Micrococcales* | *Micrococcaceae* | *Pseudarthrobacter* | - |
| 81 | *Enterobacter* sp.AO-3 (MN720565) | *Proteobacteria* | *Gammaproteobacteria* | *Enterobacterales* | *Enterobacteriaceae* | *Enterobacter* | - |
| 82 | *Pseudarthrobacter* sp AO-4 (MN720566) | *Actinobacteria* | *Actinobacteria* | *Micrococcales* | *Micrococcaceae* | *Pseudarthrobacter* | - |
| 83 | *Pseudomonas aeruginosa* strain YL（KJ765709） | *Proteobacteria* | *Gammaproteobacteria* | *Pseudomonadales* | *Pseudomonadaceae* | *Pseudomonas* | - |
| 84 | *Acidithiobacillus thiooxidans* strain JJU-1（KM101109） | *Proteobacteria* | *Acidithiobacillia* | *Acidithiobacillales* | *Acidithiobacillaceae* | *Acidithiobacillus* | - |
| 85 | *Novosphingobium subterraneum* strain DSM 12447（NZ_JRVC01000000） | *Proteobacteria* | *Alphaproteobacteria* | *Sphingomonadales* | *Sphingomonadaceae* | *Novosphingobium* | - |
| 86 | *Pseudomonas fluorescens* SBW25 (CAD88192) | *Proteobacteria* | *Gammaproteobacteria* | *Pseudomonadales* | *Pseudomonadaceae* | *Pseudomonas* | - |
| 87 | *Pseudomonas. fluorescens* Pf0-1 | *Proteobacteria* | *Gammaproteobacteria* | *Pseudomonadales* | *Pseudomonadaceae* | *Pseudomonas* | - |
| 88 | *Acidithiobacillus. ferrooxidans* ATCC 23270(ACK80970.1) | *Proteobacteria* | *Acidithiobacillia* | *Acidithiobacillales* | *Acidithiobacillaceae* | *Acidithiobacillus* | - |
| 89 | *Pseudomonas* sp. ZH1(MK990007) | *Proteobacteria* | *Gammaproteobacteria* | *Pseudomonadales* | *Pseudomonadaceae* | *Pseudomonas* | - |
| 90 | *Pseudomonas* sp. ZH2(MK990008) | *Proteobacteria* | *Gammaproteobacteria* | *Pseudomonadales* | *Pseudomonadaceae* | *Pseudomonas* | - |
| 91 | *Pseudomonas* sp. ZH3(MK990009) | *Proteobacteria* | *Gammaproteobacteria* | *Pseudomonadales* | *Pseudomonadaceae* | *Pseudomonas* | - |
| 92 | *Pseudomonas* sp. ZH4(MK990010) | *Proteobacteria* | *Gammaproteobacteria* | *Pseudomonadales* | *Pseudomonadaceae* | *Pseudomonas* | - |
| 93 | *Cupriavidus*.strain Dmm 5T-5-1(MG561851.1) | *Proteobacteria* | *Betaproteobacteria* | *Burkholderiales* | *Burkholderiaceae* | *Cupriavidus* | - |
| 94 | *Sulfobacillus thermotolerans* Strain Sb-K(DQ124681) | *Firmicutes* | *Clostridia* | *Clostridiales* | *Clostridiales Family XVII* | *Incertae Sedis* | - |
| 95 | *Sulfobacillus sibiricus* Strain Sb-F(AY079150) | *Firmicutes* | *Clostridia* | *Clostridiales* | *Clostridiales Family XVII* | *Incertae Sedis* | - |
| 96 | *Sulfobacillus thermosulfidooxidans* Strain Sb-S(AB089844) | *Firmicutes* | *Clostridia* | *Clostridiales* | *Clostridiales Family XVII* | *Incertae Sedis* | - |
| 97 | *Roseomonas rhizosphaerae* YW11(PHK94618) | *Proteobacteria* | *Alphaproteobacteria* | *Rhodospirillales* | *Acetobacteraceae* | *Roseomonas* | Yes |
