# Supplementary material for: A Critical Review of Resistance and Oxidation Mechanisms of Sb-Oxidizing Bacteria for the Bioremediation of Sb(III) Pollution
Source: Front Microbiol. 2021 Sep 7;12:738596. doi: 10.3389/fmicb.2021.738596 (PMC8453088; doi:10.3389/fmicb.2021.738596)
Supplement: Supplementary file 3 [file Table_3.doc]

Tab.S3 Sb(Ⅲ) and As(Ⅲ) resistance of Sb-oxidizing bacteria by minimal inhibitory concentration tests

| Name of the Sb-oxidizing bacteria | Genbank accession number | Minimal inhibitory concentration for Sb(Ⅲ) /(mM) | Minimal inhibitory concentration for As(Ⅲ) /(mM) | Culture medium | Reference | Sample sources |
| --- | --- | --- | --- | --- | --- | --- |
| *Cupriavidus* sp. NL4 | KF264570 | >0.1 | / | CDM | (Nguyen et al.,2015) | Sediment collected in the vicinity of an antimony oxide-producing factory in Korea |
| *Comamonas* sp. NL11 | KF264577 | >0.1 | / | CDM | (Nguyen et al.,2015) |  |
| *Acinetobacter* sp. NL1 | KF264567 | >0.1 | / | CDM | (Nguyen et al.,2015) |  |
| *Acinetobacter* sp. NL12 | KF264578 | >0.1 | / | CDM | (Nguyen et al.,2015) |  |
| *Pseudomonas* sp. NL2 | KF264568 | >0.1 | / | CDM | (Nguyen et al.,2015) |  |
| *Pseudomonas* sp. NL5 | KF264571 | >0.1 | / | CDM | (Nguyen et al.,2015) |  |
| *Pseudomonas* sp.NL6 | KF264572 | >0.1 | / | CDM | (Nguyen et al.,2015) |  |
| *Pseudomonas* sp. NL10 | KF264576 | >0.1 | / | CDM | (Nguyen et al.,2015) |  |
| *Shinella* sp. strain NLS1 | KY594721 | 2 | >1 | M-CDM | (Nguyen et al.,2017) | Sb-contaminated soil in Korea |
| *Ensifer* sp. strain NLS4 | KY594722 | 2 | >1 | M-CDM | (Nguyen et al.,2017) |  |
| *Variovorax* sp. LS1 | KC294017 | 0.15 | / | CDM | (Shi et al.,2013) | Subsurface soil in XKS，China |
| *Comamonas* sp. DF1 | KC294052 | 1 | / | CDM | (Shi et al.,2013) | Daye iron mine in Hubei,China |
| *Comamonas* sp. DF2 | KC294053 | 1 | / | CDM | (Shi et al.,2013) |  |
| *Comamonas* sp. DS1 | KC294078 | 1 | / | CDM | (Shi et al.,2013) | Daye delafossite with high sulfur content in Hubei,China |
| *Comamonas* sp. JC9 | KC294047 | 0.4 | / | CDM | (Shi et al.,2013) | Jixi coal mine in Heilongjiang,China |
| *Comamonas* sp. JC12 | KC294050 | 0.3 | / | CDM | (Shi et al.,2013) |  |
| *Comamonas* sp. JC13 | KC294051 | 0.4 | / | CDM | (Shi et al.,2013) |  |
| *Acinetobacter* sp. DS2 | KC294079 | 0.5 | / | CDM | (Shi et al.,2013) | Daye delafossite with high sulfur content in Hubei,China |
| *Acinetobacter* sp. LH3 | KC294029 | 11 | / | CDM | (Shi et al.,2013) | High Sb content mine in XKS，China |
| *Acinetobacter* sp. LH4 | KC294030 | 2.5 | / | CDM | (Shi et al.,2013) |  |
| *Acinetobacter* sp. DC2 | KC294067 | 0.05 | / | CDM | (Shi et al.,2013) | Daye Tonglvshan copper mine in Hubei, China |
| *Pseudomonas* sp. DS7 | KC294084 | 7.5 | / | CDM | (Shi et al.,2013) | Daye delafossite with high sulfur content in Hubei,China |
| *Pseudomonas* sp. DF5 | KC294056 | 0.15 | / | CDM | (Shi et al.,2013) | Daye iron mine in Hubei,China |
| *Pseudomonas* sp. DF7 | KC294058 | 0.75 | / | CDM | (Shi et al.,2013) |  |
| *Pseudomonas* sp. DS4 | KC294081 | 7.5 | / | CDM | (Shi et al.,2013) | Daye delafossite with high sulfur content in Hubei,China |
| *Pseudomonas* sp. DA5 | KC294090 | 1.5 | / | CDM | (Shi et al.,2013) | Daye gold mine in Hubei,China |
| *Pseudomonas* sp. DF12 | KC294063 | 1.5 | / | CDM | (Shi et al.,2013) | Daye iron mine in Hubei,China |
| *Pseudomonas* sp. DA2 | KC294087 | 1.5 | / | CDM | (Shi et al.,2013) | Daye gold mine in Hubei,China |
| *Pseudomonas* sp. DF11 | KC294062 | 8 | / | CDM | (Shi et al.,2013) | Daye iron mine in Hubei,China |
| *Pseudomonas* sp. DF3 | KC294054 | 0.1 | / | CDM | (Shi et al.,2013) |  |
| *Pseudomonas* sp. DF9 | KC294060 | 1.5 | / | CDM | (Shi et al.,2013) |  |
| *Pseudomonas* sp. DC5 | KC294070 | 0.1 | / | CDM | (Shi et al.,2013) | Daye Tonglvshan copper mine in Hubei, China |
| *Pseudomonas* sp. DC8 | KC294073 | 7 | / | CDM | (Shi et al.,2013) |  |
| *Pseudomonas* sp. JC11 | KC294049 | 1.5 |  | CDM | (Shi et al.,2013) | Jixi coal mine in Heilongjiang,China |
| *Pseudomonas sp. TC13* | KC294138 | 0.25 | / | CDM | (Shi et al.,2013) | Coal mine in Tianjin, China |
| *Pseudomonas sp. DC7* | KC294072 | 7.5 | / | CDM | (Shi et al.,2013) | Daye Tonglvshan copper mine in Hubei, China |
| *Pseudomonas* sp. DF8 | KC294059 | 0.3 | / | CDM | (Shi et al.,2013) | Daye iron mine in Hubei,China |
| *Pseudomonas* sp. DA4 | KC294089 | 0.75 | / | CDM | (Shi et al.,2013) | Daye gold mine in Hubei,China |
| *Aminobacter sp. LS5* | KC294021 | 0.3 | / | CDM | (Shi et al.,2013) | Subsurface soil in XKS，China |
| *Paracoccus sp. LH8* | KC294034 | 0.025 | / | CDM | (Shi et al.,2013) | High Sb content mine in XKS，China |
| *Paracoccus sp. JC6* | KC294044 | 0.10 | / | CDM | (Shi et al.,2013) | Jixi coal mine in Heilongjiang,China |
| *Sphingopyxis* sp. DA6 | KC294091 | 0.05 | / | CDM | (Shi et al.,2013) | Daye gold mine in Hubei,China |
| *Sphingopyxis* sp. DS8 | KC294085 | 0.05 | / | CDM | (Shi et al.,2013) | Daye delafossite with high sulfur content in Hubei,China |
| *Bacillus subtilis sp DF4* | KC294055 | 1.5 | / | CDM | (Shi et al.,2013) | Daye iron mine in Hubei,China |
| *Janibacter limosue* LH2 | KC294028 | 11 | / | CDM | (Shi et al.,2013) | High Sb content mine in XKS，China |
| *Arthrobacter* sp. LH11 | KC294037 | 16 | / | CDM | (Shi et al.,2013) |  |
| *Acinetobacter* sp. LH3 | KC294029 | 16 | / | CDM | (Shi et al.,2013) |  |
| *Hydrogenophaga taeniospiralis* strain IDSBO-1 | KM199760 | 2 | 2 | Other | (Terry et al.,2015) | Contaminated Mine Sediments，Stibnite/Yellow Pine mining area of Idaho,USA |
| *Variovorax paradoxus* strain IDSBO-4 | KM199761 | 2 | 2 | Other | (Terry et al.,2015) |  |
| *Sulfobacillus* spp. | ~~*~~ | 100 | 9.3 | 9K | (Tsaplina et al.,2013) | Gold ores and antimony containing sulfide minerals，Russia |
| *Leptospirillum* spp | ~~*~~ | 100 | 9.3 | 9K | (Tsaplina et al.,2013) |  |
| *Ferroplasma* spp. | ~~*~~ | 100 | 9.3 | 9K | (Tsaplina et al.,2013) |  |
| *Sulfobacillus thermotolerans* Strain Sb-K | DQ124681 | 41.08 | 40.8 | 9K | (Tsaplina et al.,2010; Zhuravleva et al.,2011) | Gold_containing pyrrhotite pyrite-arsenopyrite ore with high antimony content from the Olympiadinskoe deposit，Russia |
| *Sulfobacillus sibiricus* Strain Sb-F | AY079150 | 41.08 | 40.8 | 9K | (Tsaplina et al.,2010;Zhuravleva et al.,2011) |  |
| *Sulfobacillus thermosulfidooxidans* Strain Sb-S | AB089844 | 41.08 | 40.8 | 9K | (Tsaplina et al.,2010;Zhuravleva et al.,2011) |  |
| *Ochrobactrum anthropic* TJ1 | MH345840 | 16.43 | / | Beef extract peptone AGAR medium | (Du et al.,2020) | Sb-contaminated soil in XKS，China |
| *Bacillus* sp TJ2 | MH345839 | 16.43 | / |  | (Du et al.,2020) |  |
| *Bacillus cereus* TJ3 | MH345838 | 16.43 | / |  | (Du et al.,2020) |  |
| *Comamonas* sp. S44 | FJ210285 | 0.3 | 18 | CDM-A | (Li et al.,2013;Xiong et al.,2011) | Sb-contaminated soil in XKS, China |
| *Comamonas* sp. JL25 | JF740042 | 0.4 | 10 | CDM-A | (Li et al.,2013) |  |
| *Comamonas* sp. JL40 | JF740043 | 5 | 33 | CDM-A | (Li et al.,2013) |  |
| *Variovorax* sp. JL23 | JF740055 | 1 | 13 | CDM-A | (Li et al.,2013) |  |
| *Acinetobacter* sp. JL7 | JF740033 | 2 | 20 | CDM-A | (Li et al.,2013) |  |
| *Stenotrophomonas* sp. JL9 | JF740054 | 0.1 | 12 | CDM-A | (Li et al.,2013) |  |
| *Bosea* sp. AS-1 | CP022372 | 50.2 | 57.9 | CDM | (Lu et al.,2018) | Sb-contaminated soil in XKS, China |
| *Agrobacterium* tumefaciens GW4 | AWGV00000000 | 8 | / | CDM | (Li et al.,2015) | Water sample from Shanxi,China |
| *Agrobacterium tumefaciens* A5 | AF388030 | 0.3 | / | CDM | (Hamamura et al.,2013) | Ichinokawa  Minetailing，Ehime, Japan |
| *Pseudomonas stutzeri* TS44 | EU073110 | 0.018 | 23 | LCM/HCM enrichment Medium | (Hamamura et al.,2013) |  |
| *Stenotrophomonas* *maltophilia* str. IAM 12423 | NR041577 | 0.01 | / |  | (Hamamura et al.,2013) |  |
| *Acinetobacter johnsonii* JH7 | NA565953 | 2 | / | CDM | (Gu et al.,2020) | Chashan tailings samples in Nandan County, Guangxi province, China |
| *Pseudomonas* sp.AO-1 | MN720563 | 66.1 | >0.5 | CDM-A | The author's research group | Sb-contaminated soil in XKS, China |
| *Pseudarthrobacter* sp. AO-2 | MN720564 | 41.3 | >0.5 | CDM-A |  |  |
| *Enterobacter* sp.AO-3 | MN720565 | 66.1 | >0.5 | CDM-A |  |  |
| *Pseudarthrobacter* sp AO-4 | MN720566 | 49.6 | >0.5 | CDM-A |  |  |
| *Pseudomonas* sp.ZH1 | MK990007 | >50 | / | CDM-A | (Hua et al.,2019) | Sb-contaminated soil in XKS, China |
| *Pseudomonas* sp.ZH2 | MK990008 | >50 | / | CDM-A | ( Hua et al.,2019) |  |
| *Pseudomonas* sp.ZH3 | MK990009 | >50 | / | CDM-A | ( Hua et al.,2019) |  |
| *Pseudomonas* sp.ZH4 | MK990010 | >50 | / | CDM-A | ( Hua et al.,2019) |  |
| *Cupriavidus.strain D* 5T-5-1 | MG561851.1 | >50 | / | CDM-A | ( Hua et al.,2019) |  |
| *Cupriavidus* sp. S1 | MF423089 | ~~6~~ | / | CDM | (Li et al.,2018) | Sb-contaminated soil in XKS, China8 |
| *Moraxella sp.* S2 | MF423090 | ~~8~~ | / | CDM | (Li et al.,2018) |  |
| *Bacillus* sp.S3 | MF423091 | 5.5 | / | CDM | (Li et al.,2018) |  |
| *Roseomonas rhizosphaerae YW11* | PHK94618 | 2 | 1 | Low-phosphate modified R2A medium | (Sun et al.,2020) | Korean Agriculture Culture Collection |
| *Comamonas testosteroni* S44 | ADVQ00000000 | 0.3 | 20.0 | LB | (Xiong et al.,2011) | Sb-contaminated soil in XKS, China |

**Note:** "*" in the table represents that the antimony resistance microbial are non-pure species. M-CDM, CDM-A, represent other media improved based on conventional CDM media combined with practical experiments. “Other” means other special conditions of cultivation. XKS is the abbreviation of Xikuangshan antimony deposit in Lengshuijiang, Hunan Province, SX is Shanyin County, Shanxi Province. The paper of "the author's research group" is being published and has not been published yet.
